# Transcriptome and morphological analysis on the heart in gestational protein-restricted aging male rat offspring

**DOI:** 10.3389/fcell.2022.892322

**Published:** 2022-10-24

**Authors:** Marina S. Folguieri, Ana Teresa Barufi Franco, André Schwambach Vieira, José Antonio Rocha Gontijo, Patricia Aline Boer

**Affiliations:** ^1^ Fetal Programming and Hydroelectrolyte Metabolism Laboratory, Nucleus of Medicine and Experimental Surgery, Department of Internal Medicine, FCM, Campinas, Brazil; ^2^ Department of Structural and Functional Biology, Biology Institute, State University of Campinas (UNICAMP), Campinas, Brazil

**Keywords:** fetal programming, gestational protein-restriction, cardiovascular disease, arterial hypertension, myocyte hypertrophy, heart miRNA transcriptome

## Abstract

**Background:** Adverse factors that influence embryo/fetal development are correlated with increased risk of cardiovascular disease (CVD), type-2 diabetes, arterial hypertension, obesity, insulin resistance, impaired kidney development, psychiatric disorders, and enhanced susceptibility to oxidative stress and inflammatory processes in adulthood. Human and experimental studies have demonstrated a reciprocal relationship between birthweight and cardiovascular diseases, implying intrauterine adverse events in the onset of these abnormalities. In this way, it is plausible that confirmed functional and morphological heart changes caused by gestational protein restriction could be related to epigenetic effects anticipating cardiovascular disorders and reducing the survival time of these animals.

**Methods:** Wistar rats were divided into two groups according to the protein diet content offered during the pregnancy: a normal protein diet (NP, 17%) or a Low-protein diet (LP, 6%). The arterial pressure was measured, and the cardiac mass, cardiomyocytes area, gene expression, collagen content, and immunostaining of proteins were performed in the cardiac tissue of male 62-weeks old NP compared to LP offspring.

**Results:** In the current study, we showed a low birthweight followed by catch-up growth phenomena associated with high blood pressure development, increased heart collagen content, and cardiomyocyte area in 62-week-old LP offspring. mRNA sequencing analysis identified changes in the expression level of 137 genes, considering genes with a *p*-value < 0.05. No gene was. Significantly changed according to the adj-p-value. After gene-to-gene biological evaluation and relevance, the study demonstrated significant differences in genes linked to inflammatory activity, oxidative stress, apoptosis process, autophagy, hypertrophy, and fibrosis pathways resulting in heart function disorders.

**Conclusion:** The present study suggests that gestational protein restriction leads to early cardiac diseases in the LP progeny. It is hypothesized that heart dysfunction is associated with fibrosis, myocyte hypertrophy, and multiple abnormal gene expression. Considering the above findings, it may suppose a close link between maternal protein restriction, specific gene expression, and progressive heart failure.

## Introduction

The developmental origin and mechanisms related to cardiovascular disease (CVD) are reasonably well known in human and animal models. According to the World Health Organization (World Health Organization, 2021), in humans, CVD is the leading cause of death globally, reaching 32% (17.9 million) worldwide deaths in 2019. The risk factors were generally associated with maternal intrauterine environment commitment. Barker et al. (1989) were the first to observe the inverse relationship between birth weight and CVD prevalence, interpreting the embryonic and fetal environments as a new component in the etiology of these diseases (Barker et al., 1989b). Epidemiological and experimental studies have demonstrated the intimate association between perinatal food restriction and a higher prevalence of CVD in adulthood [Langley-EvansGardner and Welham, 1998; Roseboom et al., 2000; Hinton et al., 2018; Assalin et al., 2019; Mariano et al., 2021]. From Barker’s hypothesis, Alan Lucas grounded fetal programming in 1991 (Barker et al., 1989a; Lucas, 1991). Fetal programming could be caused by psychological and nutritional stress, as well as by placental ischemia. During experimental studies of fetal/embryonic development stages, these phenomena lead to long-term adverse effects on organs structure and function with an increasing predictive chance of developing chronic disorders (Lucas, 1991; Mesquita et al., 2010a,b; Sene et al., 2013; Sene et al., 2018; Sene et al., 2021; Lamana et al., 2021), including increased risk of cardiovascular and metabolic diseases (Langley-Evans et al., 1999; Gluckman & Hanson, 2004; McMullen et al., 2005; Burdge et al., 2007; Hanson & Gluckman, 2008) in adult life. Studies in rodents have emphasized that fetal programming results in low birth weight, which is associated with an increased risk of arterial hypertension, cardiovascular disorders, and chronic renal failure in adulthood (Mesquita et al., 2010a,b; Sene et al., 2013, 2018, 2021; Lamana et al., 2021; Mariano et al., 2021; Grigoletti-Lima et al., 2022). In response to a hostile intrauterine environment, the fetus/embryo undergoes adaptations that accelerate the maturation and differentiation of tissues and organs to the detriment of cell proliferation, restricting fetus growth (Fall 2013). The mechanisms by which fetal-programmed offspring lead to a higher incidence of CVD are not fully understood. However, cardiovascular disorders probably result from progressive epigenetic events that lead to an imbalance in the development and maintenance of the cardiovascular system (Barker et al., 1989b; Lakatta, 2003; Odden et al., 2011). Prior study has suggested that heart dysfunction is also associated with enhanced heart fibrosis and myocyte hypertrophy associated with multiple miRNA expression changes (Assalin et al., 2019). In addition, the gestational commitment overlaps with age-associated factors (smoking, physical inactivity, changes in diet, and lipid metabolism), emphasizing the importance of interaction to the increased CVD incidence over the years (Gluckman & Hanson, 2004; Hu et al., 2004; McMullen et al., 2005). Therefore, prior research assumes that the LP offspring induced a precocious aging process that culminates in premature animal death (Grigoletti-Lima et al., 2022). So, it is plausible that gestational protein restriction and its effects may influence gene expression and predispose cardiovascular disorders, which in turn may impact and modify the survival curve of the LP progeny compared to NP offspring. Considering the above findings, this study evaluated a close link between maternal protein restriction, specific gene expression, progressive heart failure, and reduced survival time of these animals. Thus, we may suppose that the current study may contribute to the best understanding of the onset of cardiovascular disease in adult offspring from the mother submitted to adverse events during pregnancy.

## Material and methods

### Animals and experimental design

The experiments were conducted on male offspring originating from inbred female and male Wistar HanUnib rats (250–300 g), sibling-mated, from the Multidisciplinary Center for Biological Research in Science in Laboratory Animals—Unicamp (CEMIB) with free access to water and standard rodent chow (Nuvital, Curitiba, PR, Brazil). The general guidelines established by the Brazilian Council for Animal Experimentation (CONCEA) were followed throughout the investigation. The Institutional Ethics Committee reviewed and approved the experimental protocol (CEUA/Unicamp, protocol #4272-1, 2016) at the Campinas State University (Unicamp)—Campinas, SP, Brazil. The animals were kept in a suitable environment under 23 ± 2°C, 50 ± 10% relative humidity, and a light-dark cycle from 7:00 a.m. to 7:00 p.m. At 11 weeks of age, the animals were mated, and the day that sperm were seen in the vaginal smear was designated day one of pregnancy. Then, dams were maintained *ad libitum* throughout the entire pregnancy on an isocaloric rodent laboratory chow with either standard protein content [NP, *n* = 10] (17% protein) or low protein content [LP, *n* = 10] (6% protein) diet (Langley-EvansGardner and Welham, 1998). In the current study, we established the age of 62 weeks to perform examinations. Morphological and molecular analyses were performed in NP and LP male offspring (rats from different mothers). The NP and LP maternal food consumption were determined daily (subsequently normalized for body weight), and the bodyweight of dams was recorded weekly in both groups. Male pups from the NP (*n* = 49) and LP (*n* = 51) different litters were weighed at birth. After weaning, the offspring of both experimental groups were followed and maintained under normoprotein rodent laboratory chow weekly until the 62nd week of life. Bodyweight (BW) was obtained on the 48th, and at the 62nd week, rats were euthanized for experimental procedures. Langley-Evans & Sculley (2006), whose experimental protocol was very similar to the present one, have shown that male rodents submitted to gestational protein restriction (9%) had a mean survival (identical to the median) of 69 weeks. Based on this study, we established the ages of 48 and 62 weeks to be evaluated experimentally. In addition, the heart left (LV) and right (RV) ventricles and adipose tissue were weighed. Left ventricle samples from 62-week-old animals were collected for mRNA sequence, immunohistochemistry, and western blot.

### Blood pressure measurement

Briefly, the indirect tail-cuff blood pressure measurement method was determined by an electro-sphygmomanometer combined with a pneumatic pulse transducer/amplifier. The Windaq software graphically established the blood pressure from the Automatic Cuff Inflation Pump, ITC Life Science Inc.^®^. The systolic arterial pressure was measured in a random sample of conscious animals, gently restrained in specific containers purchased by the device manufacturer at 32, 40, 48, 52, and 62-week-old NP (*n* = 15) and compared to age-matched LP (*n* = 25) male offspring. Measurements were conducted during the same period of the day. The mean of three consecutive readings was taken as the blood pressure. This indirect approach allowed repeated measures with close correlation (correlation coefficient = 0.975) compared with direct intra-arterial recording.

### Collagen content, myocyte area, and left ventricle thickness

Male 62-week-old LP (*n* = 5) and age-matched NP (*n* = 5) offspring from different litters were anesthetized with a mixture of ketamine (50 mg/kg body weight, i.p.) and xylazine (1 mg/kg body weight, i.p.). The LV was perfused with a heparinized saline solution (1%) followed by a 4% (w/v) paraformaldehyde solution in 0.1 M phosphate buffer (pH 7.4). After perfusion, the LV was dissected, fixed for 24 h in the paraformaldehyde solution, and then embedded in the paraplast (Sigma-Aldrich, United States). Five-micrometer-thick sections were stained with hematoxylin and eosin (HE) or picrosirius red. The measurements were performed from digital images collected by a video camera attached to an Olympus microscope (× 40 magnification lens), and the images were analyzed by ImageJ software. The cross-sectional area was measured with a digital pad. Selected cells were transversely cut so that the nucleus was in the center of the myocyte and determined an average of at least 30 myocytes per animal. The left ventricular interstitial collagen volume fraction was marked by picrosirus-red and stained with a fast green was calculated; as the ratio between the connective tissue area and connective tissue plus myocyte areas from 30 microscope fields of digitalized images of each animal. Perivascular collagen was excluded from the morphological study analysis. The stained LV slides images were also scanned and analyzed using the ImageJ software to measure wall thickness and lumen area. Also, the cross-sectional myocyte slices from 62-week-old NP (*n* = 5) and LP (*n* = 5) offspring from different litters were stained with hematoxylin and eosin. The selected cells, measured with a digital pad, were transversely cut so that the nucleus was in the center of the myocyte and determined an average of at least 30 myocytes per animal. The CellSens Dimension program estimated the concentration of collagen present in the groups.

### Adiposity index

Forty-eight-week-old and 62-week-old male NP (*n* = 15) and age-matched LP (*n* = 15) progeny from different litters were anesthetized by isoflurane. The adipose tissue was then dissected and weighed. Total body fat was measured as the sum of the following individual fat pad weights: epididymal fat + retroperitoneal fat + visceral fat. The adiposity index was calculated as (total body fat/final BW) x100.

### Total RNA extraction and RNA sequencing

Based on the protocol developed and revised by Chomczynski & Sacchi (2006), LV total RNA from 62-week-old male LP (*n* = 5) offspring compared to age-matched NP progeny (*n* = 5) from different litters was extracted using the Trizol extraction method. A High Capacity cDNA reverse transcription kit (Life Technologies, United States) was used for the cDNA synthesis. Two μl cDNA (40 ng/μl) was added to containing specific primers (Table 1) and the SYBR^®^ Green JumpStartTM Taq ReadyMixTM (catalog number S4438). The total RNA quantity, purity, and integrity were assessed previously for miRNAs expression analysis (Assalin et al., 2019; Sene et al., 2013, 2018, 2021). Before sending the samples for sequencing, a library was built for each sample, following the protocol provided by the manufacturer of the TruSeq Stranded mRNA kit (Illumina) (https://www.illumina.com/products/by-type/sequencing-kits/library-prepkits/truseq-stranded-mrna.html). Sequencing was performed on the Hiseq platform at LaCTAD—Central Laboratory for High-Performance Technologies, UNICAMP, Campinas, Brazil. Reads were aligned to the *Rattus norvegicus* Ensembl Rnor 5.0 assemble genome using the STAR aligner tool.

**TABLE 1:** Primer sequences forward and reverses (5’-3’).

	Primer Fwd (5’-3’)	Primer Rev (5’-3’)
ADRB 1	CGC​TGC​CCT​TTC​GCT​ACC​AG	CCGCCACCAGTGCRGAGGAT
CBP	TGGAGAAGCAAGGAGGTC	GCG​GCG​TAA​GGA​AGA​GAA​C
IRS2	GTA​TTA​GAT​AAG​GAA​CCA​AGA​GGC	AAA​GTA​ACA​GGA​GAA​ATG​ACA​GCA
RAP 1	TGC​TTG​AAA​TCC​TGG​ATA​CTG	AGC​CTT​GTC​CGT​TCT​TCA​TGT​AC
P300	GAC​CCT​CAG​CTT​TTA​GGA​ATC​C	TGC​CGT​AGC​AAC​ACA​GTG​TCT
TXN2	CGG​ACA​TTT​CAC​ACC​ACC​AGA​G	CCG​TGC​TGT​TTG​GCT​ACC​ATC
GARDH	CCT​TCA​TTG​ACC​TCA​ACT​ACA​TG	CTT​CTC​CAT​GGT​GGA​AGA​C

(https://github.com/alexdobin/STAR, V 2.7.0). Data analysis was performed using the program for normalization and statistical analysis DESeq2 (DESeq2 package version: 1.35.0). (Data available in NCBI’s Gene Expression Omnibus, accessible through GEO Series accession number GSE188836 (https://www.ncbi.nlm.nih.gov/geo/query/acc.cgi?acc=GSE188836). Data are expressed as the mean ± standard deviation, and the estimates of dispersion and logarithmic fold changes incorporate data-driven prior distributions in NCBI’s Gene Expression Omnibus. A list of all differentially expressed genes in the treated group compared with the control group was generated. The transcriptome was analyzed using DAVID Bioinformatics Resources 6.8—Laboratory of Human Retrovirology and Immunoinformatics (LHRI) (https://david.ncifcrf.gov/). Genes with significantly altered expression, assuming *p* < 0.05, were shown in the current study and analyzed for their function and possible action pathways. Functional analysis was performed based on pertinent literature on aging and cardiac disease. Gene function was investigated based on published articles from peer-review indexed journals available in scientific databases by search engines such as PubMed (www.ncbi.nlm.nih.gov/pubmed/) and Web of Science (https://apps.webofknowledge.com) (Supplementary Table S1).

The RNA-Seq validation was performed using cDNA analysis for real-time PCR assays (RT-qPCR). The PCR cycles were performed in the StepOne Plus equipment (Life Technologies, United States) under the following conditions: 94°C for 2 min, followed by 40 cycles of 94°C for 15 s and 60°C for 1 min. Ct values were converted to relative expression values using the ΔΔCt method. The offspring heart data were normalized to GAPDH (Glyceraldehyde 3-phosphate dehydrogenase) used as endogenous control and reference gene.

Immunoblotting—LV of 62-week-old male NP (*n* = 5) and LP (*n* = 5) offspring from different litters were used to perform the protein level analysis by western blot. LV was homogenized in solubilization buffer (100 mM Tris-hydroxymethyl-aminomethane pH7.4, 10 mM sodium pyrophosphate, 100 mM sodium fluoride, 10 mM ethylenediaminetetraacetic acid, 10 mM sodium vanadate, 2 mM phenylmethylsulfonyl fluoride and 0.1 mg/ml aprotinin) using a polytron PTA 20 S generator (model PT 10/35) Brinkmann Instruments, Westbury, N.Y. United States) at maximum speed. The tissue extracted was incubated with 10% volume Triton-X 100 and then centrifuged at × 22.050 g at 4°C for 40 min. Supernatant proteins were quantified using the Biuret method. The samples were mixed with Laemmli buffer containing 100 mM dithiothreitol, heating at 95°C for 5 min. Each sample (120 ug of protein) was subjected to gel electrophoresis in Bio-Rad mini gel apparatus (Mini-Protean SDS-Page, Bio-Rad Laboratories, Hercules, C.A. United States). The proteins separated in the gel were transferred to the nitrocellulose membrane and performed for 90 min at 120 V using the BioRad^®^ mini gel electro-transfer equipment. After the transfer, the membrane was stained with Ponceau for normalization and later prepared to be immersed in the primary antibody of interest (Table 2) and, subsequently, immersed in the secondary antibody solution. Non-specific protein binding was reduced by incubating the membrane for 1 h at ambient temperature in blocking buffer (5% bovine serum albumin (BSA), 10 mM Tris, 150 mM NaCl, and 0.02% Tween 20). A Supersignal West Pico Chemiluminescent Substrate chemiluminescence kit (Thermo Scientific^®^) was used to reveal the membranes. The bands were quantified by optical densitometry using the software Un-Scan-It gel 6.1.

**TABLE 2:** List of antibodies used manufacturer and catalog number.

Antibody	Manufacturer	Catalog
AT1	Santa Cruz	Sc-31181
AT2	Santa Cruz	sc-9040
AKT	Santa Cruz	sc-8312
pAKT	Santa Cruz	sc-101629
CREB	Cell Signaling	9104
pCREB	Cell Signaling	9191
ERK1/2	Cell Signaling	4370S
Na/KATPase	Santa Cruz	sc-21713
NOS1	Santa Cruz	sc-648
SOD2	Santa Cruz	sc-30080
STAT3	Santa Cruz	sc-483
pSTAT3	Santa Cruz	sc-8001
AMPK	Cell Signaling	25321
pAMPK	Cell Signaling	25351
JAK2	Santa Cruz	sc-278
pJAK2	Cell Signaling	3775

### Data analysis

Data was previously tested to assess the normality of distribution frequency and equality of variance by the Shapiro-Wilk and the Levene test. Data are expressed as the mean ± standard deviation. Comparisons between two groups were performed using Student’s t-test when data were normally distributed and the Mann-Whitney test when distributions were non-normal. Comparisons between two groups through the weeks were performed using 2-way ANOVA for repeated measurements test, in which the first factor was the protein content in the pregnant dam’s diet and the second factor was time. The mean values were compared using Tukey´s post hoc analysis when the interaction was significant. Significant differences in the transcriptome were detected using a moderated *t*-test. Data analysis was performed with Sigma Plot v12.0 (SPSS Inc. Chicago, IL, United States). The significance level was 5%.

## Results

**FIGURE 1:**
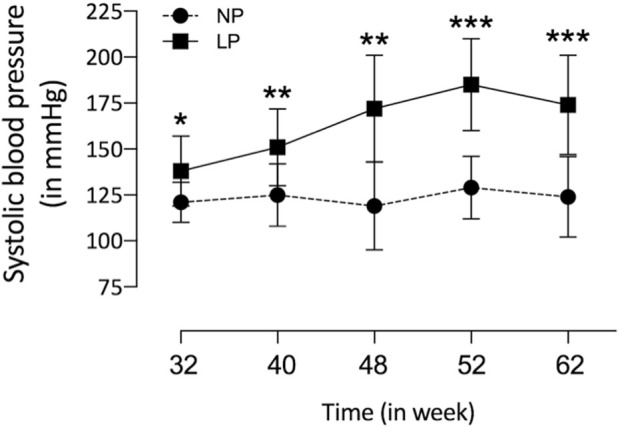
Tail systolic arterial pressure from NP (*n* = 15) compared to age-matched LP (*n* = 25) male offspring from 32nd to 62nd weeks of age was measured by an indirect tail-cuff method using an electrosphygmomanometer. The results were expressed as mean ± SD, and the two-way ANOVA statistical analysis was performed. *p < 0.05 **p < 0.01, ***p < 0.001.

**FIGURE 2:**
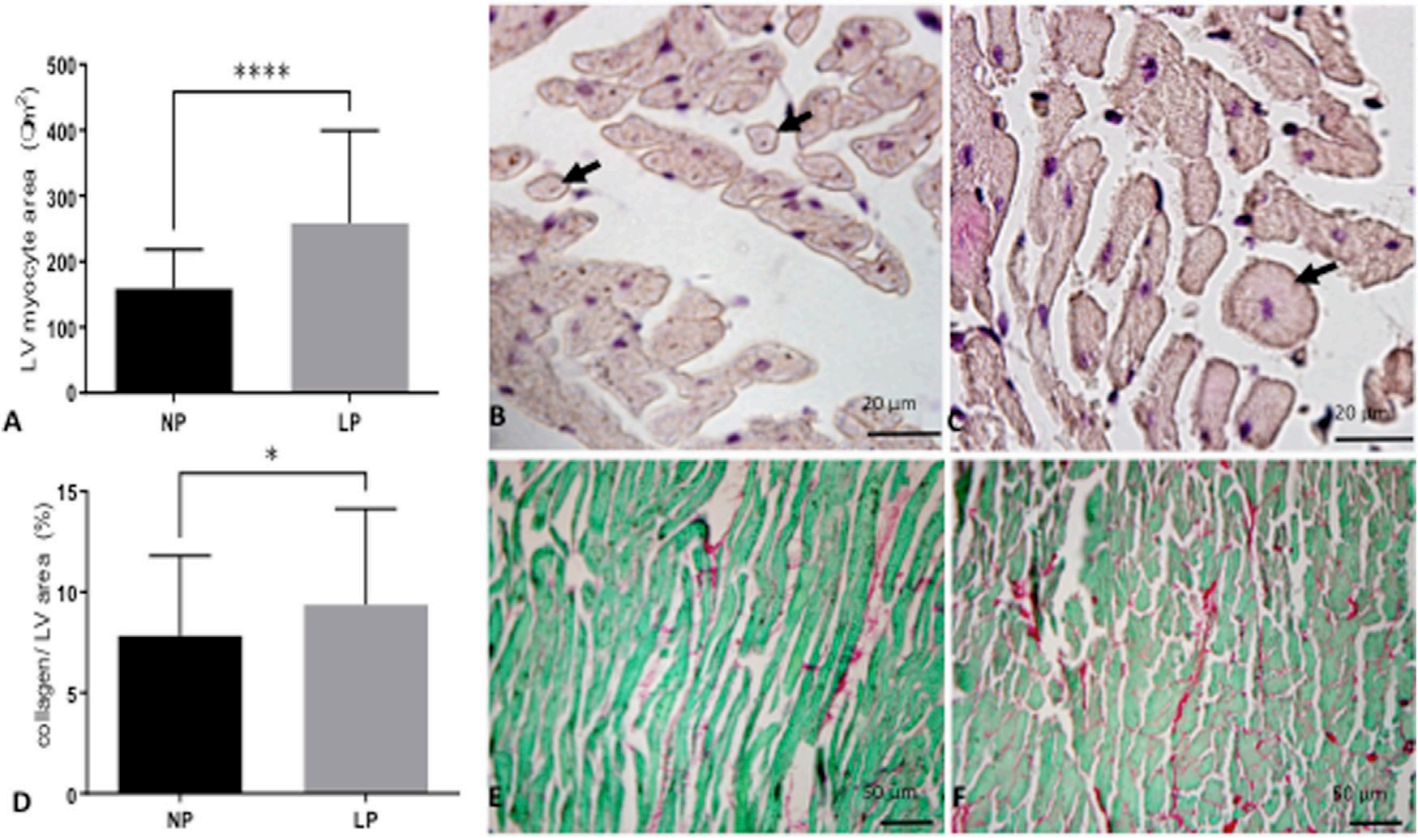
In A, a graphic of LV cardiomyocytes (arrow) area (NP: 174 fields; LP: 199 fields from *n* = 5) and representative micrographs of NP offspring **(B)** and age-matched LP progeny **(C)** LV tissue stained with hematoxylin-eosin (HE). In D, depicted LV collagen content (100 fields for each group from *n* = 5) and picrosirius fast green stained LV tissue from NP **(E)** and LP **(F)** offspring. The results were expressed as mean ± SD, and Student’s t-test performed the statistical analysis. *p < 0.05, ****p < 0.0001.

The dam’s body masses were not different in the LP compared to NP groups during the whole three weeks of gestation (1st wk: LP: 276 ± 25 vs. NP 267 ± 25; 2nd wk: LP: 303 ± 26 vs. NP 290 ± 29; 3rd wk: LP: 325 ± 37 vs. NP 325 ± 29). The birth weight of male LP progeny (*n* = 51, from 10 different mothers) was significantly smaller than that observed in male NP (*n* = 49, from 10 other mothers) offspring (LP: 6 ± 0.6 vs. NP: 6.4 ± 0.7, *p* = 0.0028). After 7 days from delivery, the LP and NP offspring body masses are similar (LP: 15.3 ± 2 vs. NP: 15.8 ± 1.6, *p* = 0.1, *n* = 35). As shown in Figure 1, the systolic arterial pressure was significantly higher in LP than NP offspring beyond 48 weeks (*p* = 0.001) until the tend experimental time. At 48 weeks of age, the body mass of LP (*n* = 51) and NP (*n* = 49) progenies were also equal (Table 3). However, at 62 weeks of age, the LP offspring showed lower body mass than the NP rats (Table 3). The heart, RV, and LV masses from LP (*n* = 15) were higher than in NP (*n* = 15) offspring at 62 weeks of age (Table 3). The LP offspring presented a significantly enhanced adiposity index (*n* = 15) when compared to those found in the NP group (*n* = 15) at 48 weeks of age (Table 3). At 62 weeks of age, the adiposity index from LP (*n* = 15) was unchanged compared to NP (*n* = 15) progeny (*p* = 0.7) (Table 3). The LV lumen plotted area was not altered in LP; however, the heart wall thickness was significantly enhanced at 62-week-old LP (Table 3) compared to age-matched NP offspring. The plotted area of cardiac myocyte and the immunostained collagen content increases significantly in the 62-week-old LP (LP: 9.364 ± 4.7 in % of collagen per LV area, *n* = 97) progeny compared to the age-matched NP (NP: 7.830 ± 3.982 in % of collagen per LV area, n = 96, *p* = 0.0303) (Figure 2).

**TABLE 3:** The table depicts the progeny's bodyweight, heart and left (LV), right ventricles (RV) masses, adiposity index, LV lumen, and wall measures.

	48 weeks		62 weeks	
	NP	LP	NP	LP
Animal mass (g)	567	583	633 ± 37	580 ± 42***
Heart/animal mass x 100 (g)	0.19 ± 0.001	0.19 ± 0.03	0.18 ± 0.01	0.2 ± 0.01**
RV/animal mass x 100 (g)	0.039 ± 0.003	0.038 ± 0.01	0.03 ± 0.006	0.035 ± 0.006*
LV/animal mass x 100 (g)	0.15 ± 0.0001	0.15 ± 0.02	0.15 ± 0.01	0.16 ± 0.01*
Adiposity index x 100	5.6 ± 1.5	5.9 ± 2.5*	6.5 ± 1.5	6.4 ± 1.6
LV lumen (pixel3)	—	—	0.03 ± 0.008	0.03 ± 0.005
LV wall thickness (pixel3)	—	—	0.065 ± 0.008	0.075 ± 0.007*

Bodyweight (BW) was obtained on the 48th and 62nd week progeny (NP; *n*=49) and (LP; *n*=51) from different litters,For heart, RV, LV masses, and adiposity (*n*=15 for each experimental group). LV lumen and wall thickness (*n*=5 for each experimental group). Comparisons betweeen experimental were performed using 2-way ANOVA for repeated measurements or Student’s test, *p< 0.05, ***p< 0.01, ***p< 0.001

**FIGURE 3:**
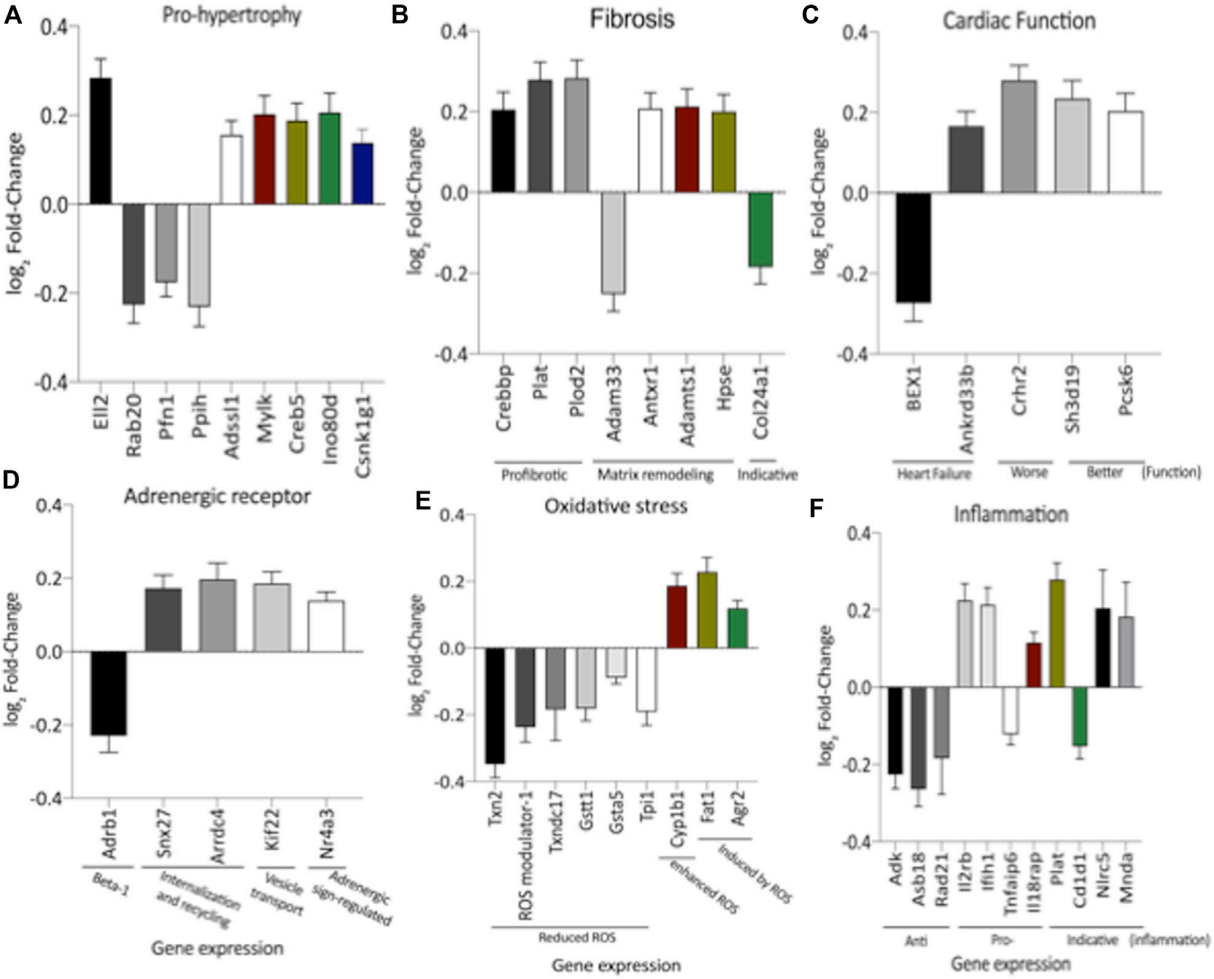
Expression of genes significantly altered, grouped by their hole in hypertrophy **(A)**, interstitial fibrosis **(B)**, cardiac function **(C)**, adrenergic receptor signaling **(D)**, oxidative stress **(E)** and inflammatory activity **(F)**. This analysis was performed based on relevant literature on aging and cardiac disease (the gene’s abbreviations and references are found in Supplementary Table S1). The results were expressed as mean ± SEM.

The literature findings categorize genes implicated in cardiac function using differentially expressed genes identified by transcriptome analysis (Supplementary Tables S1,S2). These specified genes expression correlated with cardiac function, hypertrophy, fibrosis, inflammation, oxidative stress, and adrenergic receptors (Supplementary Tables S1,S2; Figure 3). However, when the current study analyzed the LV global transcriptome, the LP (*n* = 5) progeny presented 137 differentially expressed genes (*p* < 0.05) compared to NP (*n* = 5) offspring being that 13 of these had unknown transcripts Supplementary Tables S1,S2). Supplementary Figures S1,S2 depicted the PCA plot to illustrate the samples’ relatedness and a volcano plot representing the magnitude of changes in RNA-Seq.

**FIGURE 4:**
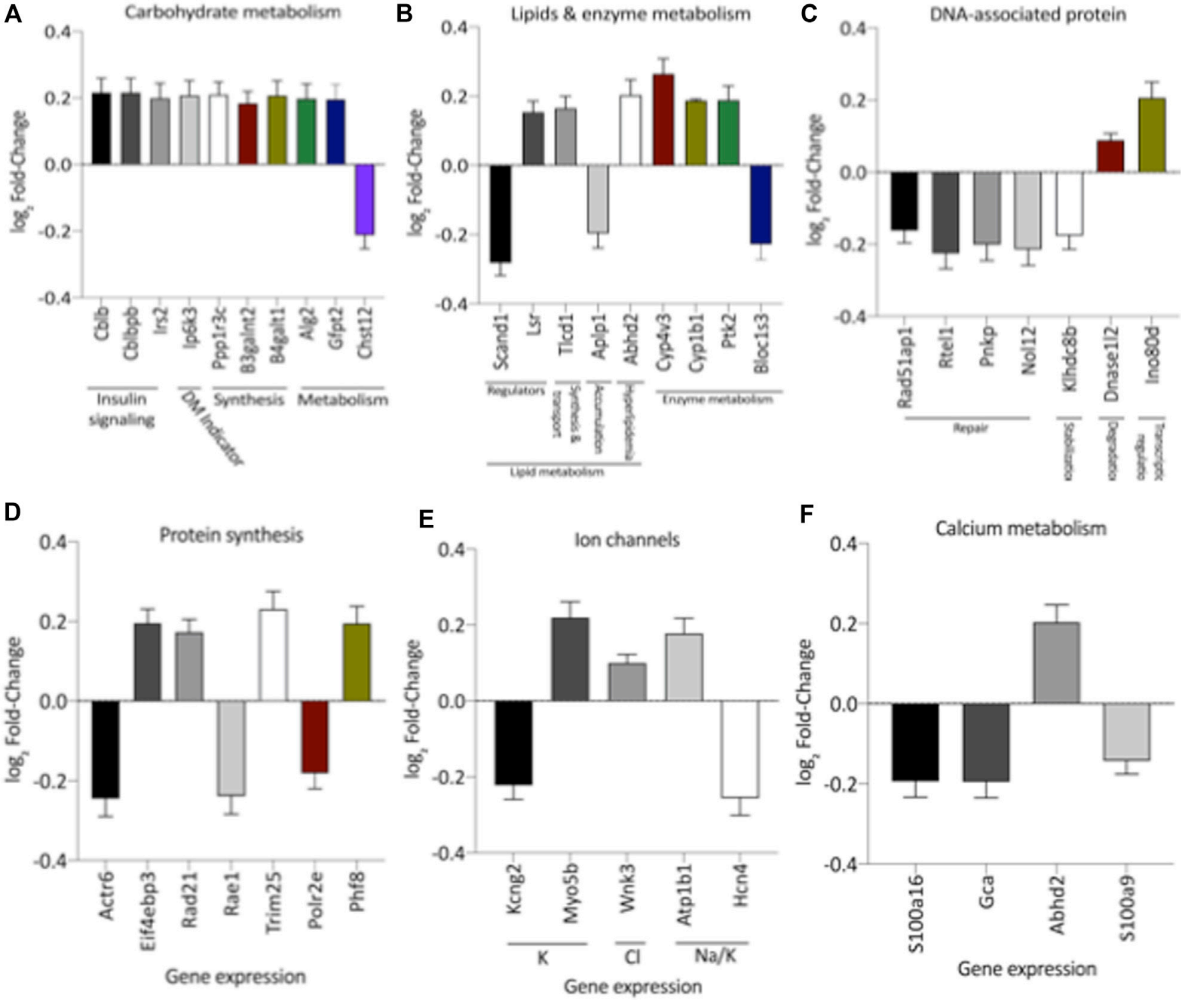
Expression of genes significantly, grouped by their role altered in carbohydrate **(A)**, lipids metabolism **(B)**, DNA-associated protein **(C)**, protein synthesis **(D)**, and Ion **(E)** and Calcium Channels **(F)**. These analyzes were performed based on pertinent literature on aging and cardiac disease (the gene's abbreviations and references are found in Supplementary Table S1). The results were expressed as mean ± SEM.

**FIGURE 5:**
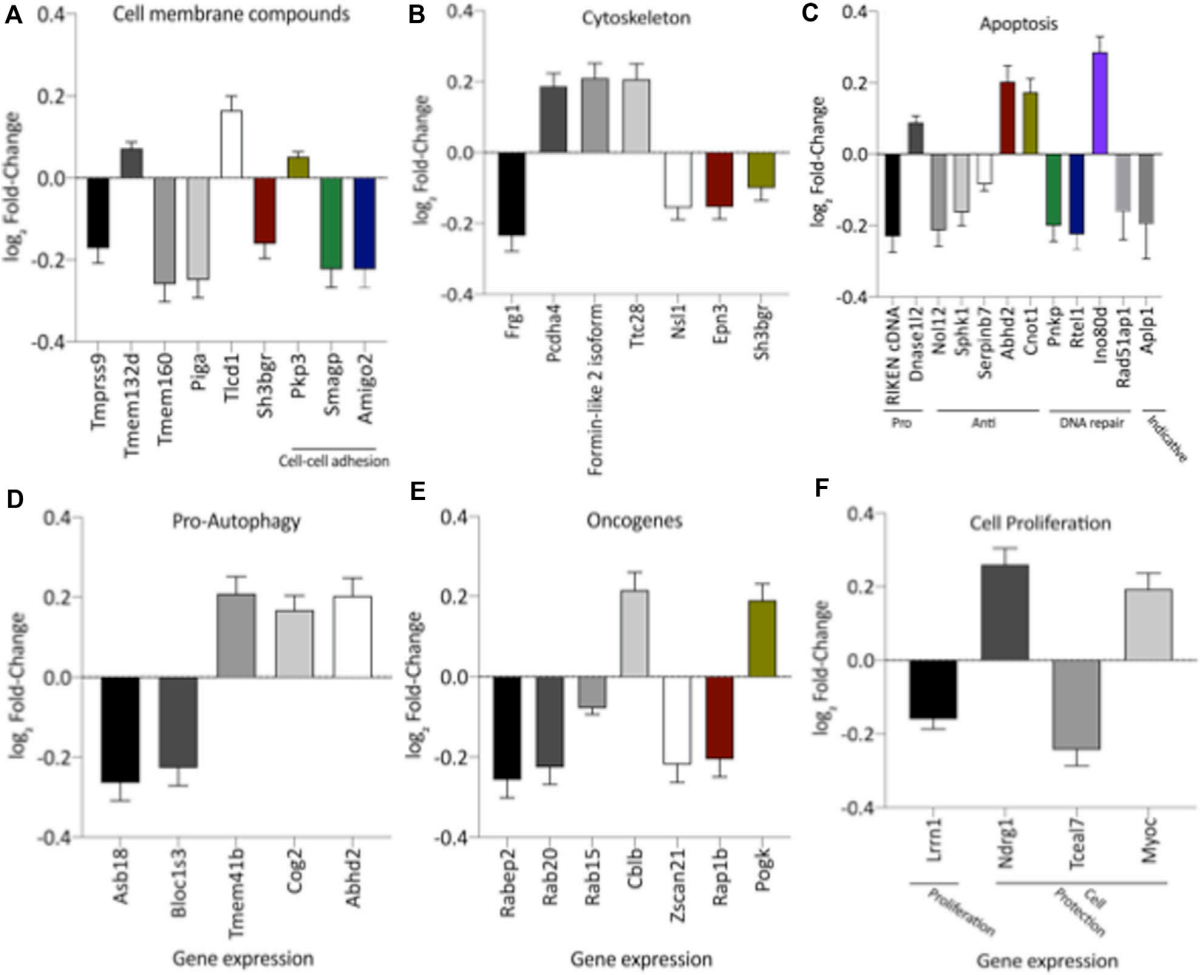
Expression of genes significantly altered, grouped by their participation in cell membrane compounds **(A)**, cytoskeleton **(B)**, apoptosis **(C)**, autophagy **(D)**, oncogenes **(E)**, and cell proliferation **(F)**. The analysis was performed based on pertinent literature on aging and cardiac disease (the gene's abbreviations and references are found in Supplementary Table S1). The results were expressed as mean ± SEM.

**FIGURE 6:**
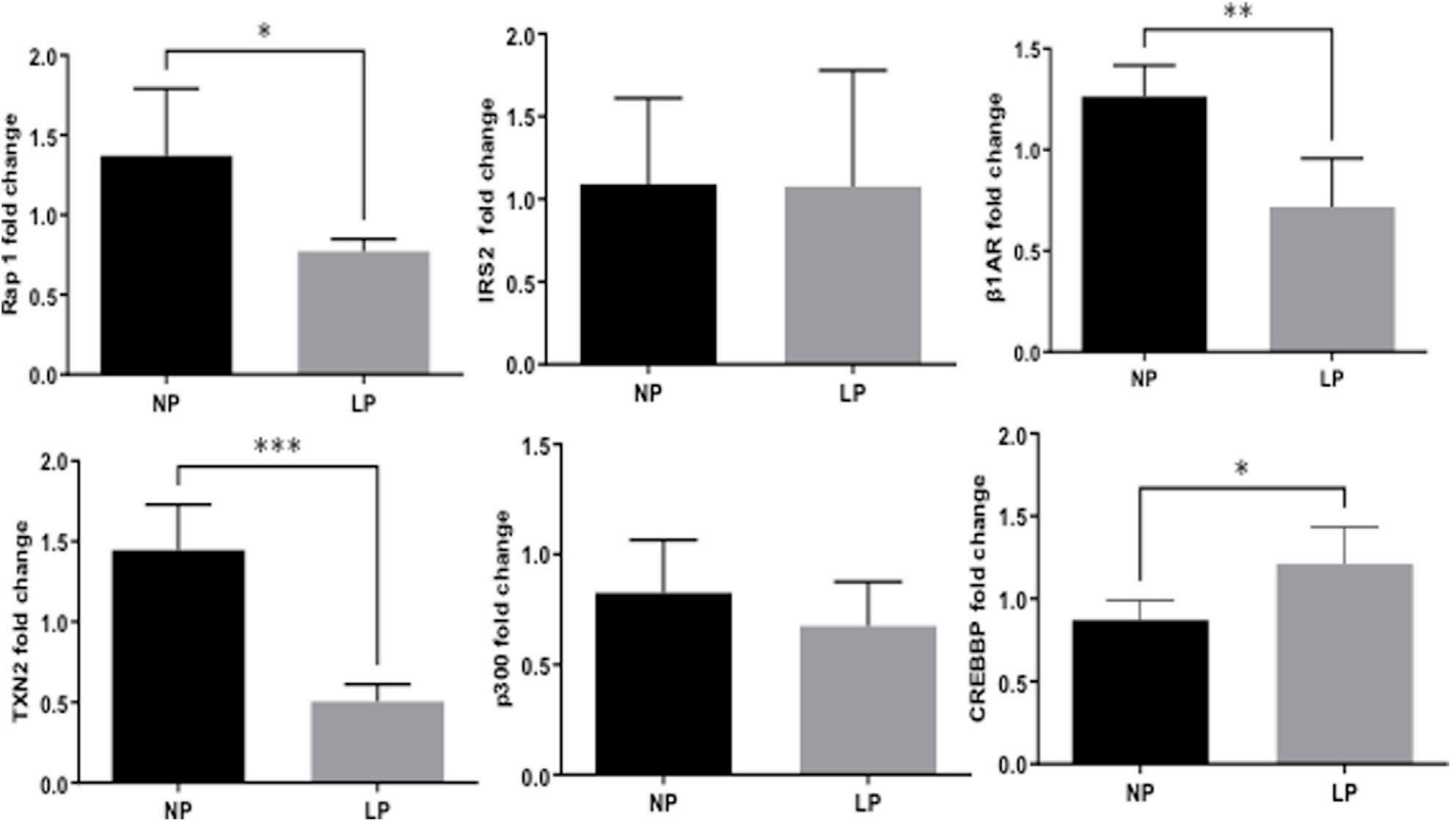
Fold-change of mRNA expression in LP offspring (*n* = 5) compared to age-matched NP progeny (*n* = 5).

**FIGURE 7:**
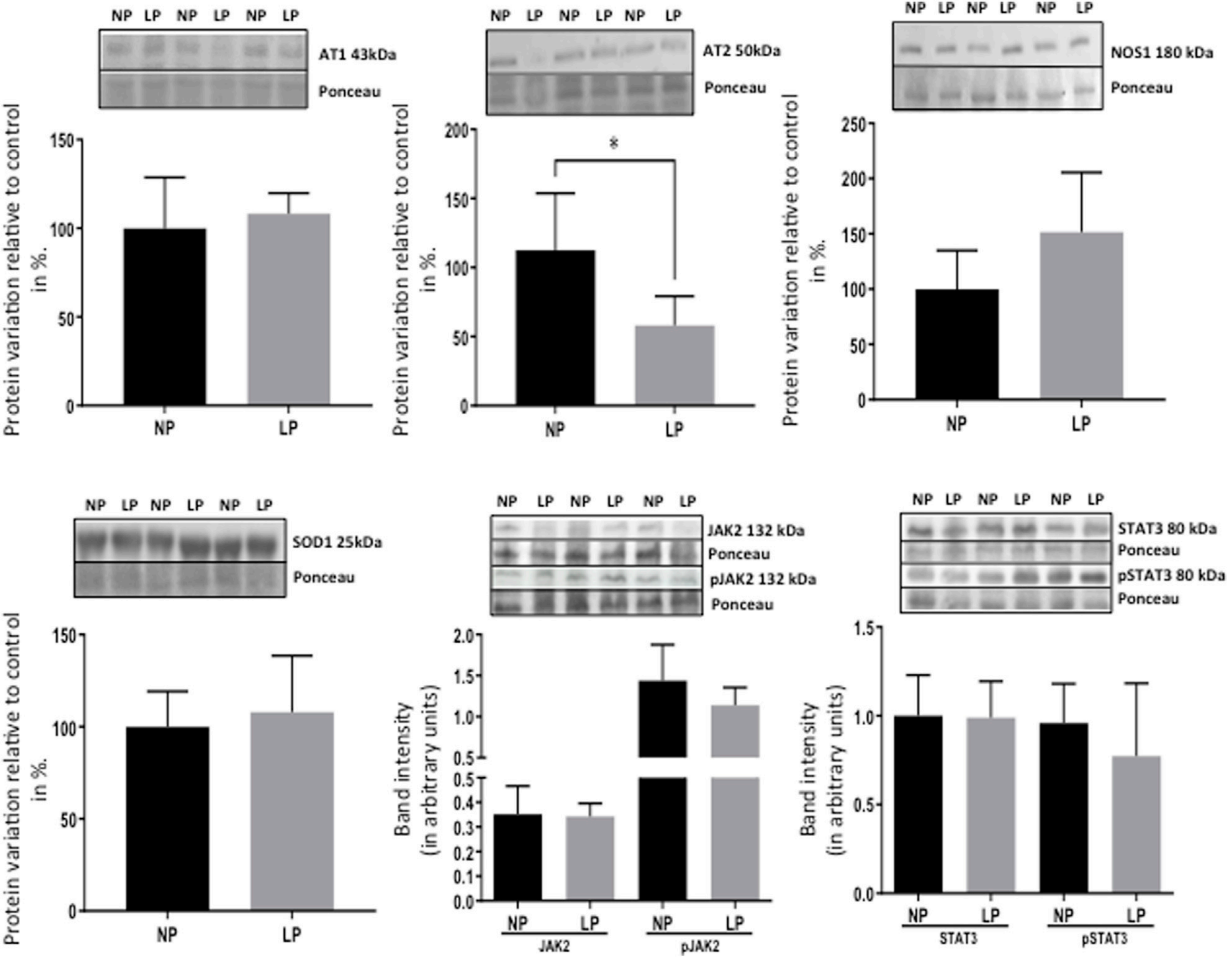
Effect of maternal protein restriction on AT1, AT2, NOS1, SOD2, JAK2, pJAK2, STAT3, and pSTAT3 proteins, measured using immunoblotting analysis in the isolated LV. The protein variation results from LP progeny were expressed as mean ± SD and relative to control (NP offspring), assigning a value in %). The statistical analysis was performed by Student’s t-test when comparing only two samples of independent observations; only one offspring of each litter was used for immunoblotting experiments. *p < 0.05.

**FIGURE 8:**
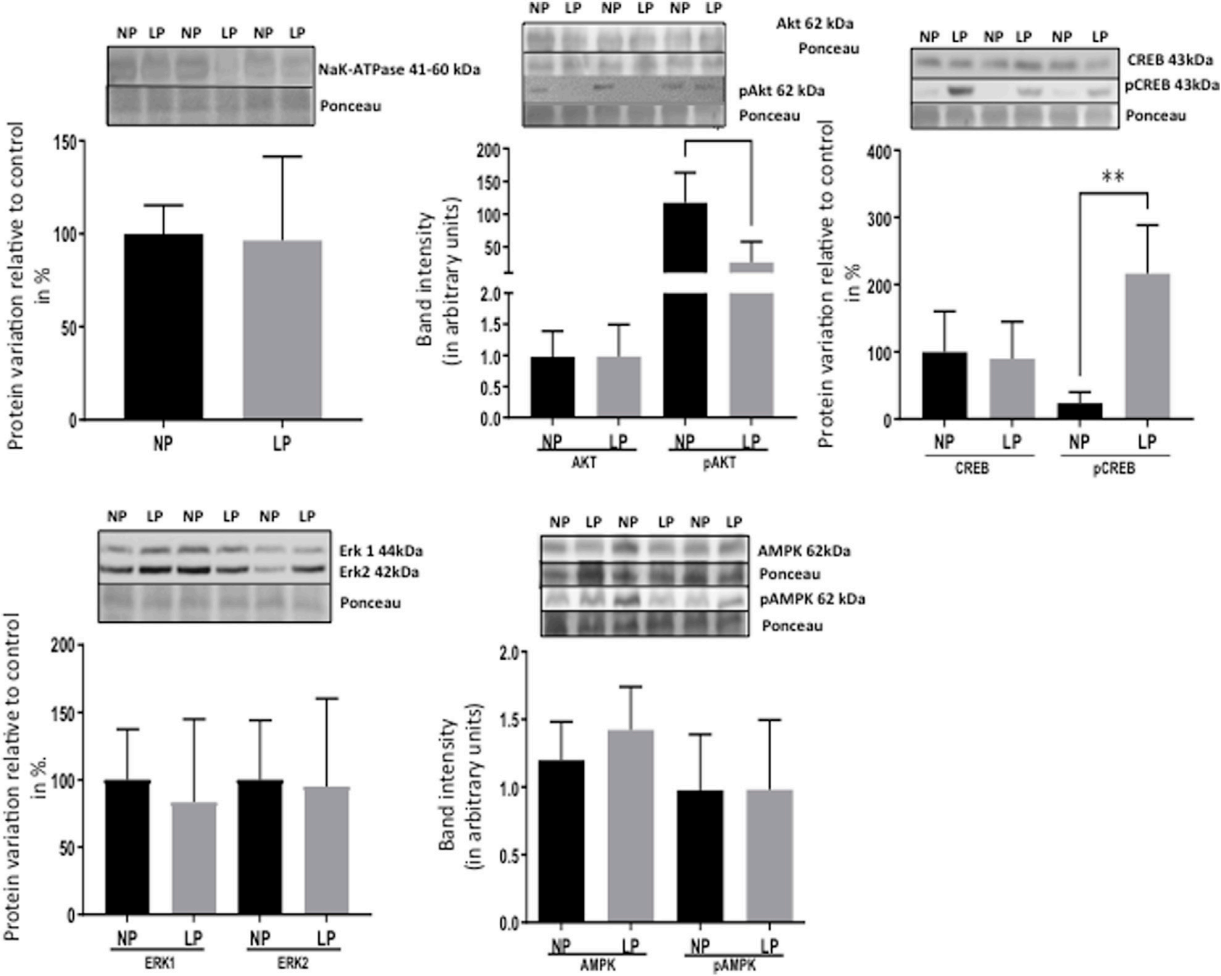
Effect of maternal protein restriction on ERK 1/2, Na-K ATPase, AKT, pAKT, CREB, pCREB, AMPk, and pAMPk proteins, measured using immunoblotting analysis in the isolated LV. The statistical analysis was performed by Student’s t-test when comparing only two samples of independent observations; only one offspring of each litter was used for immunoblotting experiments. The protein variation results from LP progeny were expressed as mean ± SD and relative to control (NP offspring), assigning a value in %. *p < 0.05; **p < 0.01.

Remarkably, the altered thioredoxin 2 (TXN2) RNA sequencing was 22% downregulated in the LP group compared to NP. Furthermore, as shown in Figure 3, significant changes in 8 other mRNAs related to oxidative stress pathways were identified. Also, they are related to insulin resistance, metabolism of carbohydrates, lipid, and enzymes (Supplementary Table S1; Figure 4), apoptosis, DNA structure, autophagy, calcium metabolism, oncogenes (Supplementary Tables S1,S2; Figure 5), protein synthesis, cell membrane compound, ion channels, and cytoskeleton (Supplementary Tables S1,S2; Figure 5). The present study validated that most mRNAs of the cAMP signaling pathway and ADRB1, CREBBP, and RAP1 were statistically different when comparing LP progeny and age-matched NP offspring (Figure 6) with no change in IRS2 and p300 expression. The investigated blotting nitric oxide synthases (NOS1), and superoxide dismutase-2 (SOD2) LV myocyte levels did not demonstrate any change in NP and LP offspring for these proteins (Figure 7); however, although not statistically significant (*p* = 0.55), NOS1 content was higher in LP animals. Regarding the cAMP and oxidative stress pathways cross-talking, despite the increase in mRNAs encoding CREB, the encoding *β*-adrenergic receptor is significantly decreased. However, the investigated blot levels of phosphorylated CREB were elevated considerably in LP offspring than in NP progeny (Figure 8). Concerning other proteins that mediate CREB expression, the study found unchanged ERK-1 and 2. However, a significant reduction in phosphorylated AKT was observed in LP progeny (Figure 8).

**FIGURE 9:**
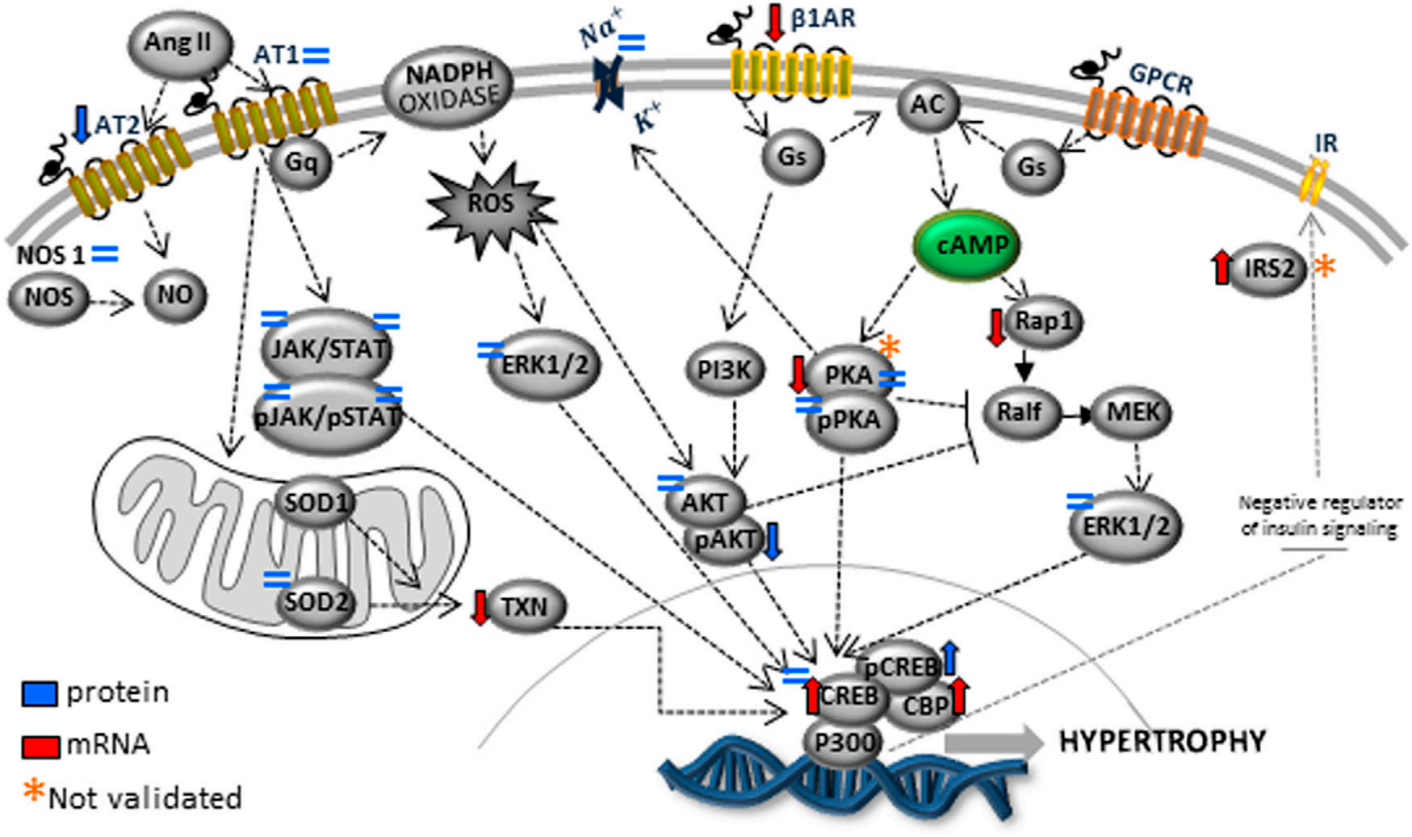
The picture depicted a schematic representation of the cyclic AMP and associated pathways investigated and biological response disorders in LV from 62nd weeks of age male rats from maternal restricted-protein intake.

The present study also studied the expression of the Ang-II receptors. Despite not observing significant changes in AT1, the expression of the AT2 receptor was considerably reduced in LP progeny (Figure 7), resulting in a substantial enhancement in AT1/AT2 ratio. Also, the current finding observed a significant reduction of the mRNA encoding PKA, which positively regulates the expression of Na/K-ATPase in LP compared to the NP group. However, the increased Na-K ATPase transcript expression in gestational protein-restricted offspring was not confirmed by an immunostaining study of the content of this sodium pump in heart tissue of animals with 62-week of life compared to age-matched controls (Figure 8). Figure 9 depicted a schematic representation of the cyclic AMP and associated pathways investigated and biological response disorders in LV from 62nd weeks of age male rats from maternal restricted-protein intake. The cAMP-signaling path (Figure 9) was recognized by transcriptome analysis using DAVID Bioinformatics Resources 6.8—Laboratory of Human Retrovirology and Immunoinformatics (LHRI) (https://david.ncifcrf.gov/). The data discussed in this publication have been deposited in NCBI’s Gene Expression Omnibus and are accessible through GEO Series accession number GSE188836 (https://www.ncbi.nlm.nih.gov/geo/query/acc.cgi?acc=GSE188836).

## Discussion

The current study confirms previous data showing that gestational protein restriction is associated with reduced embryo/fetal growth and low birth weight compared to NP offspring (Edwards et al., 2001; Fernandez-Twinn, 2004; Corstius et al., 2005; Mesquita et al., 2010a, Mesquita et al., 2010b; De Lima et al., 2013; Lamana et al., 2021). However, the LP progeny mass body recovered from one week of life, with no significant difference related to age-matched NP progeny. The rapid body mass gain is known as catch-up growth (Cianfarani et al., 1999; Zohdi et al., 2015), a well-established additional risk factor for several diseases in adult life (Ong et al., 2000; Forsén et al., 2000; Law et al., 2002; Tarry-Adkins et al., 2009). This effect leads to gender-related disorders in blood pressure, glucose metabolism, and anxiety-like behaviors in male adult progeny compared to female offspring (Kwong et al., 2000; Gillette et al., 2017). Also, it is essential to state here that sex hormones determine sexual phenotype dimorphism in the fetal-programmed disease model in adulthood by changes in the long-term control of neural, cardiac, and endocrine functions. Specific hormones and the estrus cycle interfere with behavioral, hemodynamic, and systemic water and ion homeostasis in female rodents. Thus, the present study was limited and performed on male rats considering the findings above to eliminate interferences due to gender differences (Kwong et al., 2000; Gillette et al., 2017). However, additional research with long-term follow-up and cross-fostering, including behavioral tests in female offspring, would help specify the nature of some protein-restriction effects. Previous studies have demonstrated that the multifactorial onset of the blood pressure increase in LP offspring begins at the 8th week of life, remaining elevated beyond the 16th week of age (Edwards et al., 2001; Mesquita et al., 2010a; Mesquita et al., 2010b; Mariano et al., 2021). As confirmed in the present study, data from the 32nd to the 62nd week of life showed that the arterial pressure in the LP progeny remained consistently higher in LP offspring than in NP progeny. The increased blood pressure in LP offspring may respond to several factors such as the reduced nephron number, increased tubular Na-K ATPase expression, and sympathetic renal nerve activity, phenomena associated consequently with decreasing urinary sodium excretion and elevated blood pressure (Mesquita et al., 2010a,b; Lamana et al., 2021; Custódio et al., 2017; Mariano et al., 2021). The reduced nephron number promotes blood overflow and glomerular hyperfiltration in the remaining nephrons. These effects, in turn, naturally promote glomerular fibrosis and senescence, keeping the progressive cycle of blood pressure enhancement (Langley-Evans et al., 1999; Vehaskari et al., 2001; Lucas et al., 2001).

Data available in the literature are controversial regarding cardiac mass data. In the current study, 62-week-old LP offspring confirmed a significant reduction in body mass, in parallel with a reciprocal increase in the heart and isolated ventricles weight when normalized by animals’ body mass. The discrepancy in results from prior studies may be related to using different animal strains, protein restriction levels, and the duration and growth time when the protein restriction was implemented (Zohdi et al., 2015). So, studies with Wistar Kyoto lineage subjected to protein restriction during the whole pregnancy period cause a significant reduction in heart mass (Corstius et al., 2005). Still, the dams of Wistar Kyoto rats, maintaining restricted protein intake during breastfeeding, showed uneven results, characterized by unchanged or increased heart mass compared to control offspring (Lim et al., 2010; Jackson et al., 2002). A restricted protein intake (6% protein) prior study in our laboratory demonstrated an increased left ventricular mass and heart volume in 16-week-old LP offspring (Silva et al., 2013; Assalin et al., 2019). However, the present study did not confirm the enhanced cardiac mass in 62-week-old LP progeny.

Studies have demonstrated that enhanced collagen content, in turn, reduces myocardial elasticity and compliance, causing decreased contractility and, consequently, myocardial dysfunction (Capasso et al., 1990; Burlew & Weber, 2002; Assalin et al., 2019). It has been observed that collagen is the main structural protein in the extracellular matrix, providing support and rigidity to the myocardium (Xu et al., 2006). Its deposition increases with aging (Weber, 1989; Eghbali et al., 1988). In 16-week old gestational protein-restricted progeny, we showed a striking increase in the cardiomyocyte cross-sectional area associated with enhanced interstitial collagen deposition in the LV (Assalin et al., 2019). In the present study, evaluating the ventricle collagen content in 62-week-old LP progeny, a significant enhancement deposition was also found (Lim et al., 2006; Xu et al., 2006; Menendez-Castro et al., 2011; Menendez-Castro et al., 2014; Assalin et al., 2019). Additionally, we demonstrated significant hypertrophy of cardiomyocytes in 62-week-old LP offspring, as previously observed by other authors (Morrison et al., 2007; Bubb et al., 2007; Louey et al., 2007). This finding can be taken as an immediate adaptive myocardial remodeling process in response to pressure overload, humoral factors, or compensatory mechanisms resulting in a contraction deficit (Sohal et al., 1993; Tarry-Adkins et al., 2010; He et al., 2015). Notably, previous studies confirming our data have demonstrated that gestational protein restriction reduces the number of cardiomyocytes (Corstius et al., 2005; Xu et al., 2006).

We may also assume that the hypertrophied cardiomyocytes may result from arterial hypertension or compensatory mechanisms for temporarily maintaining adequate cardiac output in 62-week-old LP offspring. In the current study, the reduced myocyte number may attenuate striking an expected enhanced heart mass in the LP offspring despite the cardiomyocyte hypertrophy.

CREB is a transcription factor responsible for activating genes in response to external factors that lead to cardiac hypertrophy and fibrosis (Kumar & Pandey, 2009). Regarding the cAMP signaling pathway, a significant increase in the expression of mRNAs encoding CREB5 and CREB binding protein (CREBBP) was observed in 62-week-old LP progeny (Figure 3). However, two of the mRNAs encoding CREB pathway meditators showed a significant decrease (PKA and Rap1) in LP offspring compared to the NP group. By blot analysis, the current study showed a substantial increase in phosphorylated CREB in LP animals, suggesting that additional pathways besides the cAMP could activate CREB expression.

The current study analyzing type 1 and 2 Ang-II receptors establishes that an increased AT1/AT2 ratio causes receptor imbalance, prevailing an enhanced type 1 Ang-II receptor stimuli, leading to increased CREB phosphorylation. Also, changes in renin-angiotensin-system (RAS) components characterized by raised renal angiotensin AT1/AT2 receptors ratio expression could explain the high blood pressure compared to NP rats (Mesquita et al., 2010a,b). By the way, prior studies have demonstrated that activation of type-1 Ang-II receptors leads to CREB phosphorylation throughout a signaling pathway involving PI3K/AKT and Erk1/2, which results in cardiac hypertrophy and associated fibrosis (Cammarota et al., 2002; Sahar et al., 2007; Shanmugam et al., 2011). However, considering mediators involved in CREB phosphorylation, the present data did not show significant ERK1/2 protein blot detection change. Thus, we may suppose that increased phosphorylated CREB could be associated with non-adaptive hypertrophy, fibrosis, and reduction in the heart myocytes, presumably by activating Type 1 Ang-II receptors phosphorylation but not by ERK1/2 and other MAPKs cascades protein expression.

Phosphorylated Akt is an essential intermediate protein in the insulin pathway activation. Otherwise, a substantial reduction in phosphorylated Akt was observed in the left ventricle of LP progeny compared to age-matched NP. We may suppose that the considerable increase in mRNA encoding Cblb (Casitas B-lineage lymphoma b), an ubiquitin E3 ligase that specifically degrades IRS1, may also be involved in pAkt reduction (Nakao et al., 2009). Also, inositol hexakisphosphate kinase-3 (IP6K3) generates inositol pyrophosphates that regulate several cellular functions. Its expression is increased in the skeletal muscle of diabetic mice. It was also observed that the IP6K3 deletion extended mice’s life span, leading authors to suggest a new role for this protein in metabolic control and survival lifetime (Moritoh et al., 2014). Here, it was demonstrated that in LV of the LP progeny, the mRNA encoding IP6K3 is significantly increased related to reduced span time of life in LP progeny compared to NP offspring, as observed in a previous study in our Lab (Grigoletti-Lima et al., 2022). In this way, cardiac aging is characterized by an increased inflammatory process and oxidative stress.

The stimulated type 1 Ang-II receptors in gestational protein-restricted offspring could further promote vasoconstriction, oxidative stress, and inflammation (Cheatham et al., 1994; Brownsey et al., 1997; Araújo et al., 2005). The assertion above is supported by changes in the expression of mRNAs encoding proteins related to increased oxidative stress and inflammation in the LP offspring compared to the NP progeny. Previous studies corroborate these findings showing that gestational protein restriction leads to greater susceptibility to oxidative stress (Langley-Evans & Sculley, 2005; Tarry-Adkins et al., 2010; He et al., 2015) and inflammatory process (Tuchscherer et al., 2012; Senna et al., 2015). In the current study, when analyzing the gene sequencing, we can observe that the most altered mRNA is the coding for Txn2, which is decreased in LP progeny. TXN2 is responsible for reducing reactive oxygen species (ROS), but we did not observe any significant difference in SOD2 expression. Although not substantial, this study also presented an increased expression of NOS1 in the LV of LP offspring. Several biological pro-inflammatory cytokines effects lead to heart failure in human and experimental models (Maier et al., 2012; Butts et al., 2015; Fenton & Parker, 2016; Deng et al., 2016). Here, in LP progeny, the LV showed the expression of four mRNAs encoding to beta-2 interleukin receptor subunit, domain 1 of interferon-induced helicase C, TNF alpha protein 6 induced, and interleukin receptor 18 accessory proteins. This data supports an inflammatory activity process in the LV of 62-week-old LP progeny. Thus, we may hypothesize that molecular effects underlying the innate immune response might be implicated in inflammation and heart disease.

The current study also demonstrated a significant reduction of mRNA encoding BEX1 (brain-expressed X-linked protein 1) in the LV of LP progeny. The expression of the BEX1 gene is linked to heart failure and is associated with gene expression related to heart disease (Accornero et al., 2017). So, the study also found an increased mRNA to encode CARP (cardiac ankyrin repeat protein) in the heart of LP offspring. CARP is predominantly expressed in cardiac muscle and is related to cardiomyocyte hypertrophy before the development of heart failure. A significant increase in CARP mRNA and protein expression in left ventricular tissue patients with end-stage heart failure is observed (Zou et al., 1997; Zolk et al., 2002).

In the heart, G protein-coupled receptors (GPCRs) respond to extracellular stimuli and are involved in fibrosis and cardiac dysfunction (Tsuda et al., 2017). *β*-Adrenergic and Ang-II receptor antagonists are used to treat patients with chronic heart failure long-term. In the current sequencing study, a reduction of β1-adrenergic receptors was observed and may be associated with increased receptor internalization and recycling, which occurs through clathrin vesicles being degraded (Von Zastrow & Kobilka, 1992; Tolbert & Lameh, 1996; Zhang et al., 1998).

Studies have demonstrated that LP offspring showed enhanced glycemia and insulinemia after the glucose tolerance test and peripheral insulin resistance (Zambrano et al., 2006; Blesson et al., 2014; Blesson et al., 2016). The PI3K/AKT/mTOR signaling pathway regulates signal transduction and biological processes such as cell proliferation, apoptosis, protein synthesis, metabolism, and angiogenesis. Under normal conditions, insulin by IRS-1 tyrosine phosphorylation triggers a signaling cascade with a vasodilating and anti-apoptotic effect throughout IRS/PI3K/AKT/mTOR pathway (Gao et al., 2002). However, factors that lead to insulin resistance, such as TNFα, fatty acids, Cblb, and Crebbp, inhibit the IRS-1 down-regulation. This effect led to an inactivation of the PI3K/AKT and stimulated ERK/MAPK pathway, promoting cell hypertrophy (Brownsey et al., 1997; Sykiotis and Papavassiliou, 2001). Erhuma et al. (2007) demonstrated that prenatal exposure to a low-protein diet is a disordered regulation of lipid metabolism in the aging rat. The ERK/MAPK pathway is involved in cell growth control, and the IRS/PI3K pathway in insulin metabolism (Cheatham et al., 1994; Saltiel & Kahn, 2001; Araújo et al., 2005). As observed in the current study, we may not rule out that peripheral insulin resistance in LP offspring could be associated with significantly enhanced adiposity index compared to the NP group at 48 weeks, as observed by authors (Zambrano et al., 2006; Blesson et al., 2014; Blesson et al., 2016). However, the finding did not confirm at 62 weeks of age. The mRNA encoding the type-2 corticotrophin-releasing hormone receptor was significantly increased in LP offspring. This transmembrane protease activates the atrial natriuretic peptide, whose deficiency may contribute to the development of arterial hypertension and heart failure (Li et al., 2017). It has recently been observed that it is expressed in exacerbating chronic heart disease (Tsuda et al., 2017). Furthermore, the Corin protein transcript defects have also been observed in LP progeny. Here, an enhanced expression of the mRNA encoding PCSK6 (Proprotein convertase subtilisin/Kexin-6), a primary activator of Corin (Chen et al., 2015), was shown in 62-week-old LP offspring. However, even though the direct activator of Corin mRNA is increased in the present model, our data could not infer this protein’s participation in the LP offspring’s heart.

Once that prior study has demonstrated that gestational protein restriction leads to increased apoptosis of cardiomyocytes (Cheema et al., 2005), the present study may suggest that gene expression related to apoptosis and pro-autophagy observed here in 62-week-old LP offspring could lead to changes in cardiac functionality.

Pro-oncogenes are genes necessary for cellular homeostasis, responsible for the growth, proliferation, and survival mechanisms related to cancer presence and physiopathology (Hanahan & Weinberg, 2000). Therefore, the increase in the expression of the pro-oncogene genes could be related to cell growth in 62-week-old LP progeny. Simultaneously, several genes expression related to membrane components, Ca++ transport, ion channels, cytoskeleton, metabolism, and enzymatic functions were shown in this study. However, it is unclear to infer specific tasks since they have a variety of roles and participate in different biological processes.

A prior study in male mice submitted to gestational protein restriction (9%) has shown a mean survival lifetime of 69 weeks, compared to mothers that received a regular protein diet (18% casein), which showed an average lifetime survival of 74 weeks (Langley-Evans & Sculley, 2006). Based on these data, the age of 62 weeks for both progenies was determined as the deadline point for evaluating the heart structure, adipose tissue mass, mRNA sequence studies, and blotting data as defined in the experimental design.

The heart structural changes could be partly due to cardiac miRNA expression modulating several genes whose function is associated with cardiac morphogenesis and function. Many gene-encoding changes resulting from gestational protein restriction could be related to several visceral offspring disorders in adulthood compared to NP progeny. Those were defined either by metabolic or inflammatory activity and oxidative stress, as well as by apoptosis, autophagy, myocyte hypertrophy, interstitial fibrosis, and precocious cardiomyocyte senescence from programmed offspring. Taking into account together, they may lead to early changes in cardiac function, causing heart failure and advanced death (Figure 9). In conclusion, the deleterious cardiac repercussions in the 62-week-old LP compared to age-matched NP offspring could be related to the lower birth weight in programmed offspring, followed by the catch-up growth phenomenon.

Additionally, metabolic disorders that occur parallel to genes encoding expression may be involved in the altered adrenergic and renin-angiotensin-aldosterone compound system, oxidative stress, and inflammatory tissue deregulation. The above disorders may lead to early cardiac hypertrophy, fibrosis, and senescence. The focus of the present study is on the heart. However, we could not rule out gene expression changes that could be secondary to adaptive responses to the programming to physiologic responses of the kidney, instead of the direct programming effect of LP diet on the male offspring heart sustained across the lifespan. Thus, a more dynamic analysis would be required to reach that conclusion; hypothetically, all these phenomena would promote organ dysfunction and the premature death of LP offspring (Figure 9).

## Data Availability

The datasets presented in this study can be found in online repositories. The names of the repository/repositories and accession number(s) can be found below: Â NCBI’s Gene Expression Omnibus, accessible through GEO Series accession number GSE188836 (https://www.ncbi.nlm.nih.gov/geo/query/acc.cgi?acc=GSE188836).

## Supplementary material

SUPPLEMENTARY FIGURE S1 The picture depicted the PCA plot to illustrate the relatedness of the samples used in RNA-Seq.

SUPPLEMENTARY FIGURE S2 The picture depicted volcano plot that represents the magnitude of changes in RNA-Seq.

SUPPLEMENTARY TABLE S1 Up (↑) and down (↓) regulated genes in left ventricles of low protein rats, biological function, and references.

SUPPLEMENTARY TABLE S2 The table depicted the values obtained for the genes studied and folded changes and adjusted p-values.

## Glossary

ADRB1: adrenoceptor beta 1
AngII: angiotensin II
ANOVA: variance analysis
AT1: abrangiotensin receptor type 1
AT2: angiotensin receptor type 2
BEX1: brain-expressed X-linked protein 1
BW: body weight
CARP: cardiac ankyrin repeat protein
Cblb: casitas B-lineage lymphoma b
CEMIB: Multidisciplinary Center for Biological Research in Science in Laboratory Animals.
abrCEUA: Institutional Ethics Committee approved the experimental design on the Use of Animals
CONCEA: Brazilian Council for Animal Experimentation
CREB: cAMP-responsive element-binding
CREBBP: CREB binding protein
CVD: cardiovascular disease
Erk1/2: extracellular signal-regulated kinase1/2
GAPDH: glyceraldehyde 3-phosphate dehydrogenase
GPCR: G protein-coupled receptor
IP6K3: inositol hexakisphosphate kinase-3
IRS1: insulin receptor substrate 1
abrIRS2: insulin receptor substrate 2
LaCTAD: Central Laboratory for High-Performance Technologies
LHRI: Laboratory of Human Retrovirology and Immunoinformatics
abrLP: low-protein
LV: left ventricle
MAPK: mitogen-activated protein kinase
mTOR: mammalian target of rapamycin
Na-K **ATPase**: sodium pump
NOS1: nitric oxide synthases 1
NP: normal protein
PCSK6: proprotein convertase subtilisin/Kexin-6
PI3K: phosphoinositide 3-kinase
PKA: protein kinase A
RAP1: a member of the RAS oncogene family
RAS: renin-angiotensin-system
ROS: reactive oxygen species
RV: right ventricle
SOD2: superoxide dismutase-2
TNF: tumor necrosis factor
TXN2: thioredoxin 2
Wk: week
