# Supplementary figures and images for: Transcriptome and morphological analysis on the heart in gestational protein-restricted aging male rat offspring
Source: Front Cell Dev Biol. 2022 Oct 24;10:892322. doi: 10.3389/fcell.2022.892322 (PMC9638007; doi:10.3389/fcell.2022.892322)

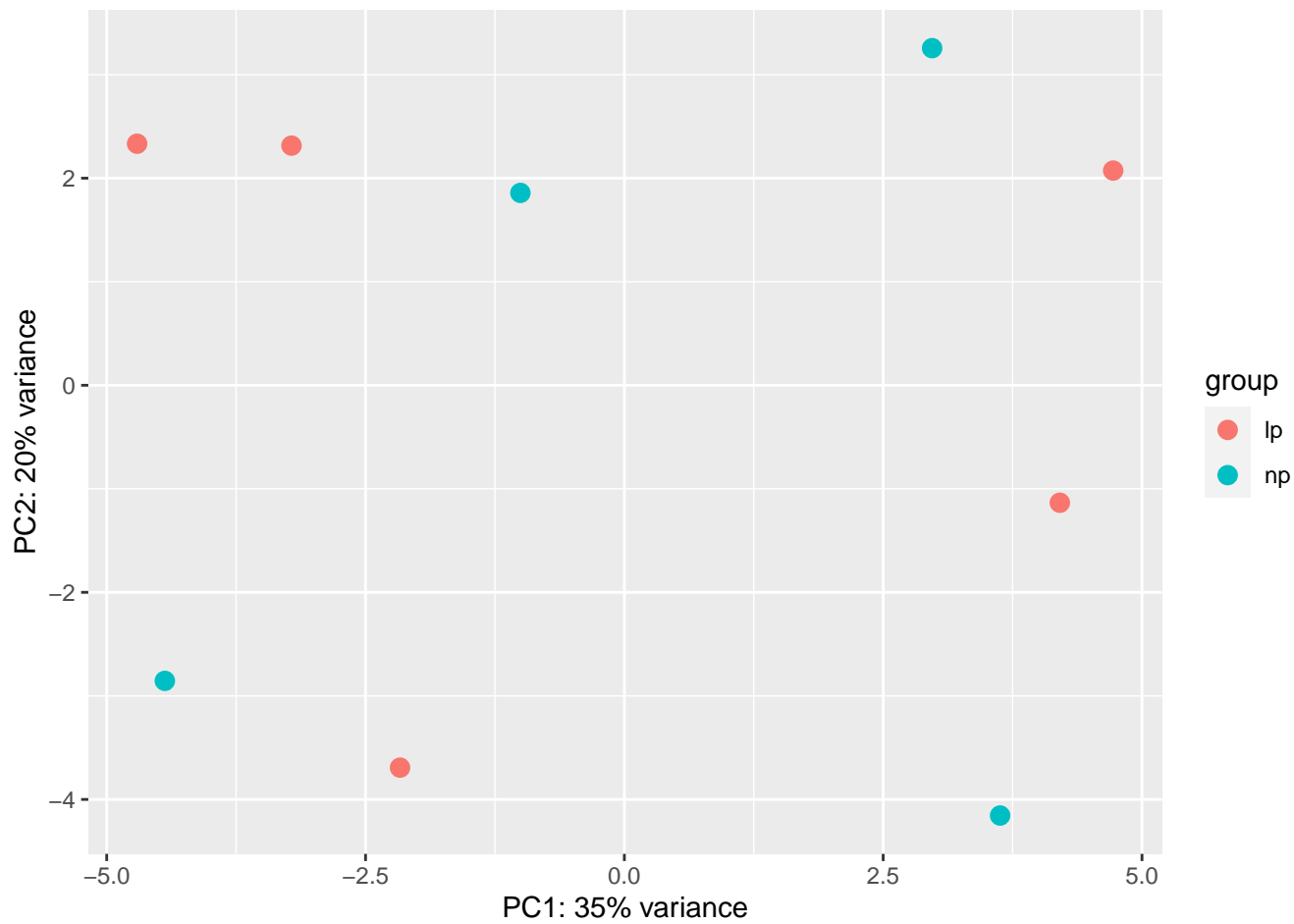

Supplement: Supplementary file 1 [file DataSheet2.PDF]

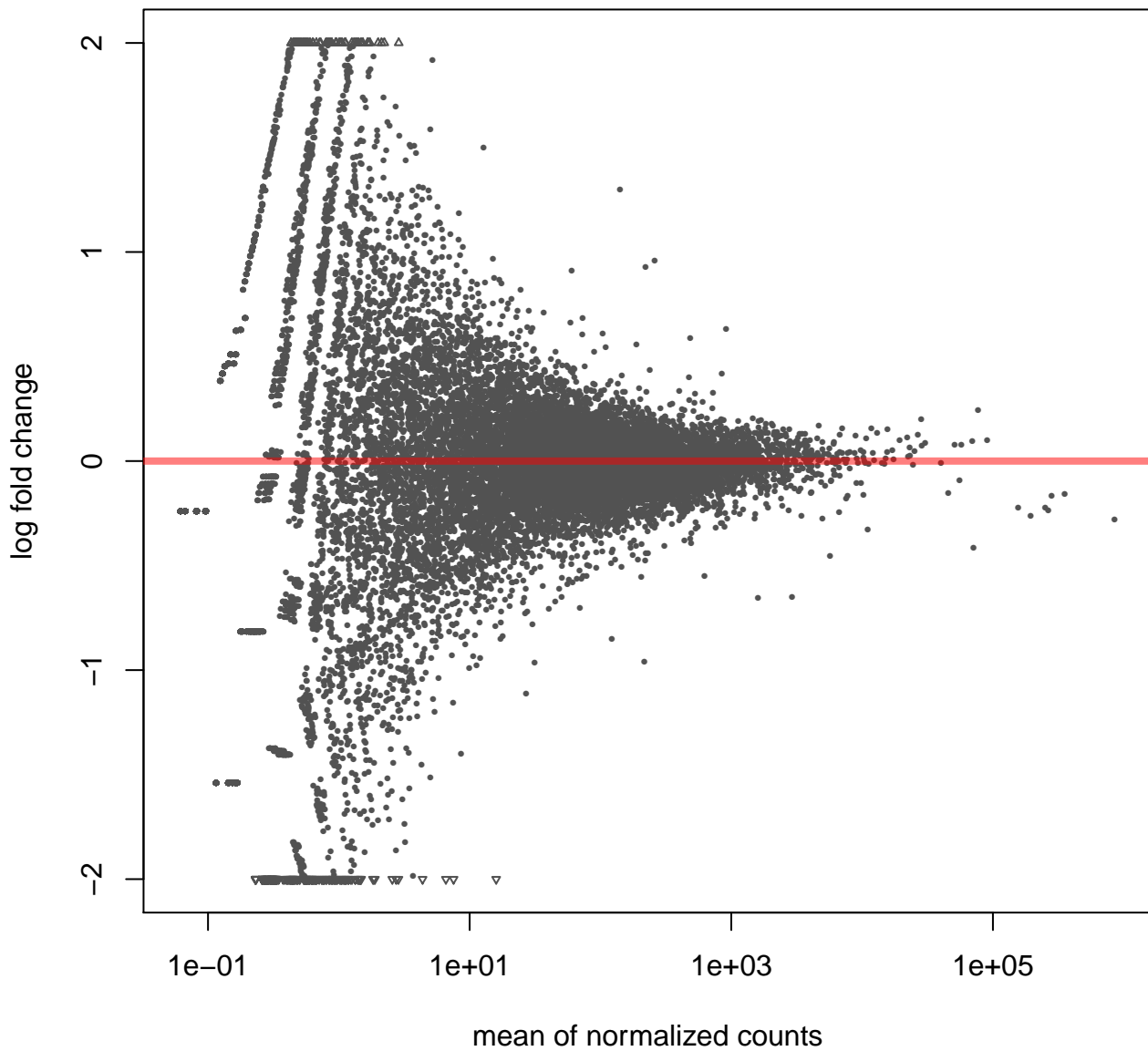

Supplement: Supplementary file 4 [file DataSheet1.PDF]
