# Supplementary material for: Transcriptome and morphological analysis on the heart in gestational protein-restricted aging male rat offspring
Source: Front Cell Dev Biol. 2022 Oct 24;10:892322. doi: 10.3389/fcell.2022.892322 (PMC9638007; doi:10.3389/fcell.2022.892322)
Supplement: Supplementary file 3 [file Table1.pdf]

Table 1S. Up (↑) and down (↓) regulated genes in left ventricles of low protein rats, biological function and references

	GENE	FUNCTION/LOCATION
**HYPERTROPHY**	↑ elongation factor for RNA polymerase II 2 (Ell2)	pro-hypertrophy (1,2)
	↓RAB20, member RAS oncogene Family (Rab20)	pro-hypertrophy (3)
	↓profilin 1(Pfn1)	pro-hypertrophy (4,5)
	↓peptidylprolyl isomerase H (Ppih)	pro-hypertrophy (6)
	↑adenylosuccinate synthase like 1 (Adssl1)	pro-hypertrophy by AngII (7)
	↑myosin light chain kinase (Mylk)	pro-hypertrophy by AngII (8)
	↑cAMP responsive element binding protein 5(Creb5)	pro-hypertrophy (9–11)
	↑INO80 complex subunit D (Ino80d)	pro-hypertrophy (12,13)
	↓casein kinase 1, gamma 1(Csnk1g1)	pro-hypertrophy (14)
	↑CREB binding protein (Crebbp)	pro-hypertrophy (11,15,16)
**FIBROSIS**	↑plasminogen activator, tissue type (Plat)	pro- fibrosis (17)
	↑procollagen lysine, 2-oxoglutarate 5-dioxygenase 2(Plod2)	pro- fibrosis (18)
	↓ADAM metallopeptidase domain 33 (Adam33)	anti- fibrosis (19)
	↑anthrax toxin receptor 1(Antxr1)	anti- fibrosis (20,21)
	↑ADAM metallopeptidase with thrombospondin type 1 motif, 1 (Adamts1)	anti- fibrosis (22)
	↑Heparanase (Hpse)	anti- fibrosis (23)
	↓collagen type XXIV alpha 1 chain (Col24a1)	fibrosis indicative (24)
**CARDIAC FUNCTION**	↓protein BEX1-like(LOC100912195) BEX1-like	increases in heart failure (25)
	↑ankyrin repeat domain 33B (Ankrd33b)	increases in heart failure (26)
	↑corticotropin releasing hormone receptor 2 (Crhr2)	worse function (27)
	↓SH3 domain binding glutamate-rich protein (Sh3bgr)	improve function (28)
	↑proprotein convertase subtilisin/kexin type 6 (Pcsk6)	improve function (29)
**ADRENORECEPTORS**	↓adrenoceptor beta 1 (Adrb1)	adrenergic receptor beta1 (30)
	↑sorting nexin family member 27 (Snx27)	internalization and recycling (31)
	↑arrestin domain containing 4 (Arrdc4)	internalization and recycling (32)
	↑kinesin family member 22 (Kif22)	cytoeskeleton (33)
	↑nuclear receptor subfamily 4, group A, member 3(Nr4a3)	regulated by adrenergic signals (34)
**INSULIN**	↑inositol hexakisphosphate kinase 3 (Ip6k3)	Increases in diabetes mellitus (35)
	↑Casitas B-lineage lymphoma b (Cblb)	irs1 degradation (36)
	↑insulin receptor substrate 2 (Irs2)	glicose metabolism (15,37)
	↑CREB binding protein (Crebbp)	Irs2 inativation (15,16)
**LIPÍDIC METABOLISM**	SCAN domain-containing 1(Scand1)	HDL metabolismo regulation (38–41)
	↑cytochrome P450, family 1, subfamily b, polypeptide 1(Cyp1b1)	lipidic metabolism (42,43)
	↑lipolysis stimulated lipoprotein receptor(Lsr)	lipidic metabolism (44)
	↑TLC domain containing 1(Tlcd1)	lipid synthesis and traffic (45–47)
	↓amyloid beta precursor like protein 1(Aplp1)	Lipid accumulation (9,48,49)
	↑abhydrolase domain containing 2(Abhd2)	hyperlipidemia marker (50)
**CARBOHYDRATE METABOLISM**	↑protein phosphatase 1, regulatory subunit 3C (Ppp1r3c)	glycogen synthesis (51)
	↑beta-1,3-N-acetylgalactosaminyltransferase 2 (B3galnt2)	oligosaccharide synthesis (52)
	↑beta-1,4-galactosyltransferase 1 (B4galt1)	carbohydrate synthesis (53)
	↑ALG2, alpha-1,3/1,6-mannosyltransferase (Alg2)	carbohydrate metabolism (54)
	↑glutamine-fructose-6-phosphate transaminase 2 (Gfpt2)	glutamine metabolism (54)
	↓carbohydrate sulfotransferase 12 (Chst12)	carbohydrate metabolism (54)
**OXIDATIVE STRESS**	↓thioredoxin 2 (Txn2)	reduces ROS (55)
	↓reactive oxygen species modulator 1-like(LOC100910944) (Modula ROS)	reduces ROS (56,57)
	↓glutathione S-transferase theta 1 (Gstt1)	reduces ROS (58)
	↓glutathione S-transferase alpha 5 (Gsta5)	reduces ROS (59)
	↓similar to Tpi1 protein(RGD1563601) (sim Tpi1)	reduces ROS (60)
	↓thioredoxin domain containing 17 (Txndc17)	reduces ROS (61)
	↑cytochrome P450, family 1, subfamily b, polypeptide 1 (Cyp1b1)	produces ROS (62)
	↑FAT atypical cadherin 1 (Fat1)	Induced by ROS (63)
	↑anterior gradient 2, protein disulphide isomerase family	Induced by ROS (64)

	member (Agr2)	
**INFLAMATION**	↓adenosine kinase(Adk)	anti-inflammation (65)
	↓ankyrin repeat and SOCS box-containing 18(Asb18)	anti-inflammation (66)
	↑RAD21 cohesin complex component(Rad21)	anti-inflammation (67)
	↑interleukin 2 receptor subunit beta(Il2rb)	pro-inflamation (68)
	↑interferon induced with helicase C domain 1(Ifih1)	pro-inflamation (69)
	↓TNF alpha induced protein 6(Tnfaip6)	pro-inflamation (70)
	↑interleukin 18 receptor accessory protein(Il18rap)	pro-inflamation (71)
	↑plasminogen activator, tissue type(Plat)	indication of inflammation (17)
	↓CD1d1 molecule(Cd1d1)	indication of inflammation (72)
	↑NLR family, CARD domain containing 5(Nlrc5)	indication of inflammation (73)
	↑myeloid cell nuclear differentiation antigen(Mnda)	indication of inflammation (74)
**APOPTOSIS**	↓similar to RIKEN cDNA B230118H07(RGD1309730) (s RIKEN cDNA)	pro- apoptosis (75)
	↑deoxyribonuclease 1 like 2 (Dnase1l2)	pro- apoptosis (76)
	↓nucleolar protein 12(Nol12)	pro- apoptosis (77)
	↓sphingosine kinase 1(Sphk1)	anti- apoptosis (78)
	↓serpin family B member 7(Serpinb7)	anti- apoptosis (79)
	↑abhydrolase domain containing 2(Abhd2)	anti- apoptosis (50)
	↑CCR4-NOT transcription complex, subunit 1(Cnot1)	anti- apoptosis (80)
	↓amyloid beta precursor like protein 1(Aplp1)	indication of apoptosis (81)
**AUTOPHAGY**	↓ankyrin repeat and SOCS box-containing 18(Asb18)	pro-autiphagy (82)
	↓biogenesis of lysosomal organelles complex-1, subunit 3(Bloc1s3)	pro-autiphagy (83)
	↑transmembrane protein 41B(Tmem41b)	pro-autiphagy (84)
	↑component of oligomeric golgi complex 2(Cog2)	pro-autiphagy (85)
	↑abhydrolase domain containing 2(Abhd2)	pro-autiphagy (50)
**ONCOGENES**	↓rabaptin, RAB GTPase binding effector protein 2(Rabep2)	oncogene (54)
	↓RAB20, member RAS oncogene family(Rab20)	oncogene (54)
	↓RAB15, member RAS oncogene family(Rab15)	oncogene (54)
	↓zinc finger and SCAN domain containing 21(Zscan21)	oncogene (54)
	↓RAP1B, member of RAS oncogene family(Rap1b)	oncogene (54)
	↑pogo transposable element with KRAB domain(Pogk)	oncogene (54)
**Ca^{+} METABOLISM**	↓S100 calcium binding protein A16(S100a16)	Ca^{++}metabolism (54)
	↓grancalcin(Gca)	Ca^{++}metabolism (54)
	↑abhydrolase domain containing 2(Abhd2)	Ca^{++}metabolism (54)
	↓S100 calcium binding protein A9(S100a9)	Ca^{++}metabolism (54)
**PROTEIN SYNTHESIS**	↓ARP6 actin-related protein 6 homolog(Actr6)	protein synthesis (86)
	↑eukaryotic translation initiation factor 4E binding protein 3(Eif4ebp3)	mRNA translation (54)
	↓ribonucleic acid export 1(Rae1)	protein synthesis (54)
	↑tripartite motif-containing 25(Trim25)	RNA ubictinization (5)
	↑INO80 complex subunit D(Ino80d)	transcriptional regulation and DNA repair (54)
	↓RNA polymerase II subunit E(Polr2e)	protein synthesis (54)
	↑PHD finger protein 8(Phf8)	protein synthesis (87)
**DNA RELATED PROTEINS**	↓polynucleotide kinase 3'-phosphatase (Pnkp)	DNA repair (88)
	↓regulator of telomere elongation helicase 1 (Rtel1)	DNA repair (89)
	↑INO80 complex subunit D (Ino80d)	DNA repair (90)
	↓RAD51 associated protein 1 (Rad51ap1)	DNA repair (91)
	↓nucleolar protein 12 (Nol12)	DNA repair (77)
	↓kelch domain containing 8B (Klhdc8b)	DNA stability (92)
	↑deoxyribonuclease 1 like 2 (Dnase1l2)	DNA stability (76)
**MEMBRANE COMPOUNDS**	↓transmembrane protease, serine 9(Tmprss9)	membrane structure (54)
	↑transmembrane protein 132D(Tmem132d)	membrane structure (54)
	↓transmembrane protein 160(Tmem160)	membrane structure (54)
	↑phosphatidylinositol glycan anchor biosynthesis, class A(Piga)	membrane structure (54)
	↑TLC domain containing 1(Tlcd1)	membrane structure (54)
	↓SH3 domain binding glutamate-rich protein(Sh3bgr)	membrane structure (54)
	↑plakophilin 3(Pkp3)	membrane structure (54)
	↓small cell adhesion glycoprotein(Smagp)	membrane structure (54)
	↓adhesion molecule with Ig like domain 2(Amigo2)	membrane structure (54)
	↓FSHD region gene 1(Frg1)	cytoskeleton (54)
	↑protocadherin alpha 4(Pcdha4)	cytoskeleton (54)

**CYTOSKELETON**	↑similar to formin-like 2 isoform B(RGD1560248) (s formin-like 2B)	cytoskeleton (54)
	↑tetratricopeptide repeat domain 28(Ttc28)	cytoskeleton (54)
	↓NSL1, MIS12 kinetochore complex component(Nsl1)	cytoskeleton (54)
	epsin 3(Epn3)	cytoskeleton (54)
	↓SH3 domain binding glutamate-rich protein(Sh3bgr)	cytoskeleton
**ENZIME METABOLISM**	↑cytochrome P450, family 4, subfamily v, polypeptide 3(Cyp4v3)	enzyme metabolism and function (54)
	↑cytochrome P450, family 1, subfamily b, polypeptide 1(Cyp1b1)	enzyme metabolism and function (54)
	↑protein tyrosine kinase 2(Ptk2)	enzyme metabolism and function (54)
	↓biogenesis of lysosomal organelles complex-1, subunit 3(Bloc1s3)	enzyme metabolism and function (54)
	↓potassium voltage-gated channel modifier subfamily G member 2(Kcng2)	ion channel (54)
**ION CHANNELS**	↑myosin Vb(Myo5b)	ion channel (54)
	↑WNK lysine deficient protein kinase 3(Wnk3)	ion channel (54)
	↑ATPase Na+/K+ transporting subunit beta 1(Atp1b1)	ion channel (54)
	↓hyperpolarization activated cyclic nucleotide-gated potassium channel 4(Hcn4)	ion channel (54)

TABLE REFERENCES

1. Yoshikawa N, Shimizu N, Maruyama T, Sano M, Matsuhashi T, Fukuda K, et al. Cardiomyocyte-Specific Overexpression of HEXIM1 Prevents Right Ventricular Hypertrophy in Hypoxia-Induced Pulmonary Hypertension in Mice. PLoS One. 2012;7(12):1–13.
2. Quaresma AJC, Bugai A, Barboric M. Cracking the control of RNA polymerase II elongation by 7SK snRNP and P-TEFb. Nucleic Acids Res. 2016;44(16):7527–39.
3. Matsuda T, Jeong JI, Ikeda S, Yamamoto T, Gao S, Babu GJ, et al. H-Ras Isoform Mediates Protection Against Pressure Overload-Induced Cardiac Dysfunction in Part Through Activation of AKT. Circ Hear Fail. 2017;10(2):1–10.
4. Kooij V, Viswanathan MC, Lee DI, Rainer PP, Schmidt W, Kronert WA, et al. Profilin modulates sarcomeric organization and mediates cardiomyocyte hypertrophy. Cardiovasc Res. 2016;110(2):238–48.
5. Choudhury NR, Heikel G, Trubitsyna M, Kubik P, Nowak JS, Webb S, et al. RNA-binding activity of TRIM25 is mediated by its PRY/SPRY domain and is required for ubiquitination. BMC Biol. 2017;15(1):1–20.
6. Toko H, Konstandin MH, Doroudgar S, Ormachea L, Joyo E, Joyo AY, et al. Regulation of Cardiac Hypertrophic Signaling by Prolyl Isomerase Pin1. Circ Res. 2014;112(9):1244–52.
7. Wen HY, Xia Y, Young ME, Taegtmeyer H, Kellems RE. The adenylosuccinate synthetase-1 gene is activated in the hypertrophied heart. J Cell Mol Med. 2002;6(2):235–43.
8. Wang S, Cheng M, Hu Z, Hu S, Zou Q, Lai X, et al. Angiotensin II Facilitates Matrix Metalloproteinase-9-Mediated Myosin Light Chain Kinase Degradation in Pressure Overload-Induced Cardiac Hypertrophy. Cell Physiol Biochem. 2018;44(6):2281–95.
9. Mahmood E, Jeganathan J, Feng R, Saraf M, Khabbaz K, Mahmood F, et al. Decreased PGC-1α Post-Cardiopulmonary Bypass Leads To Impaired Oxidative Stress In Diabetic Patients. Ann Thorac Surg [Internet]. 2018;1–10.
10. Sahar S, Reddy MA, Wong C, Meng L, Wang M, Natarajan R. Cooperation of SRC-1 and p300 with NF-κB and CREB in angiotensin II-induced IL-6 expression in vascular smooth muscle cells. Arterioscler Thromb Vasc Biol. 2007;27(7):1528–34.
11. Chien PTY, Lin CC, Hsiao L Der, Yang CM. C-Src/Pyk2/EGFR/PI3K/Akt/CREB-activated pathway contributes to human cardiomyocyte hypertrophy: Role of COX-2 induction. Mol Cell Endocrinol [Internet]. 2015;409:59–72.
12. Jiang DS, Zhang XF, Gao L, Zong J, Zhou H, Liu Y, et al. Signal regulatory protein-α protects against cardiac hypertrophy via the disruption of toll-like receptor 4 signaling. Hypertension. 2014;63(1):96–104.

13. Sanglard LP, Nascimento M, Moriel P, Sommer J, Ashwell M, Poore MH, et al. Impact of energy restriction during late gestation on the muscle and blood transcriptome of beef calves after preconditioning. BMC Genomics [Internet]. 2018;19(1):702.

14. Kusuda J, Hirai M, Tanuma R, Hashimoto K. Cloning, expression analysis and chromosome mapping of human casein kinase 1 gamma 1 (CSNK1G1): Identification of two types of cDNA encoding the kinase protein associated with heterologous carboxy-terminal sequences. Cytogenet Cell Genet [Internet]. 2000;90:298–302.

15. Peng J, He L. REVIEW IRS posttranslational modifications in regulating insulin signaling. 2018;(Guo).

16. Ghosh AK, Varga J. The Transcriptional Coactivator and Acetyltransferase p300 in Fibroblast Biology and Fibrosis. J Cell Physiol. 2007;203(3):663–71.

17. Ridker PM, Vaughan DE, Manson J, Hennekens C, Stampfer M. Endogenous tissue-type plasminogen activator and risk of myocardial infarction. Lancet. 1993;341(8854):1165–8.

18. Gjaltema RAF, De Rond S, Rots MG, Bank RA. Procollagen lysyl hydroxylase 2 expression is regulated by an alternative downstream transforming growth factor β-1 activation mechanism. J Biol Chem. 2015;290(47):28465–76.

19. Miller AC, Boldy DAR. Pulmonary embolism guidelines: Will they work? Thorax. 2003;58(6):463.

20. Besschetnova TY, Ichimura T, Katebi N, St. Croix B, Bonventre J V., Olsen BR. Regulatory mechanisms of anthrax toxin receptor 1-dependent vascular and connective tissue homeostasis. Matrix Biol [Internet]. 2015;42:56–73.

21. Hu K, Olsen BR, Besschetnova TY. Cell autonomous ANTXR1-mediated regulation of extracellular matrix components in primary fibroblasts. Matrix Biol. 2017;62:105–14.

22. Pehlivan S, Gurses MS, Ural MN, Akyol S, Eren F, Turkmen Inanir N, et al. The Role of ADAMTS1 and Versican in Human Myocardial Infarction: A Postmortem Study. Lab Med. 2016;47(3):205–12.

23. Rivara S, Milazzo FM, Giannini G. Heparanase: A rainbow pharmacological target associated to multiple pathologies including rare diseases. Future Med Chem. 2016;8(6):647–80.

24. Barnes J, Pat B, Chen YW, Powell PC, Bradley WE, Zheng J, et al. Whole-genome profiling highlights the molecular complexity underlying eccentric cardiac hypertrophy. Ther Adv Cardiovasc Dis. 2014;8(3):97–118.

25. Accornero F, Schips TG, Petrosino JM, Gu SQ, Kanisicak O, Van Berlo JH, et al. BEX1 is an RNA-dependent mediator of cardiomyopathy. Nat Commun [Internet]. 2017;8(1).

26. Zolk O, Frohme M, Maurer A, Kluxen FW, Hentsch B, Zubakov D, et al. Cardiac ankyrin repeat protein, a negative regulator of cardiac gene expression, is augmented in human heart failure. Biochem Biophys Res Commun. 2002;293(5):1377–82.

27. Tsuda T, Takefuji M, Wettschureck N, Kotani K, Morimoto R, Okumura T, et al. Corticotropin releasing hormone receptor 2 exacerbates chronic cardiac dysfunction. J Exp Med [Internet]. 2017;214(7):1877–88.

28. Jang DG, Sim HJ, Song EK, Medina-Ruiz S, Seo JK, Park TJ. A thioredoxin fold protein Sh3bgr regulates Enah and is necessary for proper sarcomere formation. Dev Biol. 2015;405(1):1–9.

29. Li H, Zhanga Y, Wu Q. Role of corin in the regulation of blood pressure. 2018;26(2):67–73.

30. Lohse MJ, Engelhardt S, Eschenhagen T. What Is the Role of β-Adrenergic Signaling in Heart Failure? Circ Res. 2003;93(10):896–906.

31. Nakagawa T, Asahi M. β1-adrenergic receptor recycles via a membranous organelle, recycling endosome, by binding with sorting Nexin27. J Membr Biol. 2013;246(7):571–9.

32. Han SO, Kommaddi RP, Shenoy SK. Distinct roles for β-arrestin2 and arrestin-domain-containing proteins in β2 adrenergic receptor trafficking. EMBO Rep [Internet]. 2013;14(2):164–71.

33. Miki H, Setou M, Kaneshiro I, Hirokawa N. All kinesin superfamily protein, KIF, genes in mouse and human. Proc Natl Acad Sci U S A [Internet]. 2001;98(13):7004–11.

34. Pearen MA, Myers SA, Raichur S, Ryall JG, Lynch GS, Muscat GEO. The orphan nuclear receptor, NOR-1, a target of β-adrenergic signaling, regulates gene expression that controls oxidative metabolism in skeletal muscle. Endocrinology. 2008;149(6):2853–65.

35. Moritoh Y, Oka M, Yasuhara Y, Hozumi H, Iwachidow K, Fuse H, et al. Inositol Hexakisphosphate Kinase 3 Regulates Metabolism and Lifespan in Mice. Sci Rep [Internet]. 2016;6(August):1–13. Available from: http://dx.doi.org/10.1038/srep32072

36. Yokoi N, Komeda K, Wang HY, Yano H, Kitada K, Saitoh Y, et al. Cblb is a major susceptibility gene for rat type 1 diabetes mellitus. Nat Genet. 2002;31(4):391–4.

37. White MF. IRS proteins and the common path to diabetes. Am J Physiol - Endocrinol Metab [Internet]. 2002;283(3):E413–22.

38. West NA, Katerina K, Dabelea D. Exposure to Maternal Diabetes in Utero and DNA Methylation Patterns in the Offspring. Immunometabolism. 2013;1:1–9.

39. Lu Y, Dollé MET, Imholz S, van 't Slot R, Verschuren WMM, Wijmenga C, et al. Multiple genetic variants along candidate pathways influence plasma high-density lipoprotein cholesterol concentrations. J Lipid Res [Internet]. 2008;49(12):2582–9.

40. Sander TL, Morris JF. Characterization of the SCAN box encoding RAZ1 gene: Analysis of cDNA transcripts, expression, and cellular localization. Gene. 2002;296(1–2):53–64.

41. Schmitz G, Heimerl S, Langmann T. Zinc finger protein ZNF202 structure and function in transcriptional control of HDL metabolism. Curr Opin Lipidol. 2004;15(2):199–208.

42. Larsen MC, Bushkofsky JR, Gorman T, Adhami V, Mukhtar H, Wang S, et al. Cytochrome P450 1B1: An unexpected modulator of liver fatty acid homeostasis. Arch Biochem Biophys [Internet]. 2015;571:21–39.

43. Bushkofsky JR, Maguire M, Larsen MC, Fong YH, Jefcoate CR. Cyp1b1 affects external control of mouse hepatocytes, fatty acid homeostasis and signaling involving HNF4α and PPARα. Arch Biochem Biophys [Internet]. 2016;597:30–47.

44. Yen FT, Roitel O, Bonnard L, Notet V, Pratte D, Stenger C, et al. Lipolysis stimulated lipoprotein receptor: A novel molecular link between hyperlipidemia, weight gain, and atherosclerosis in mice. J Biol Chem. 2008;283(37):25650–9.

45. Winter E, Chris PP. TRAM , LAG1 and CLN8 : members of a novel family of lipid-sensing domains ? TRENDSin Biochem Sci. 2002;27(8):381–3.

46. Schorling S, Vallée B, Barz WP, Riezman H, Oesterhelt D. Lag1p and Lac1p Are Essential for the Acyl-CoA – dependent Ceramide Synthase Reaction in Saccharomyces cerevisae. Mol Biol Cell. 2001;12:3417–27.

47. Ruiz M, Bodhicharla R, Svensk E, Devkota R, Boren J, Pilon M. Membrane fluidity is regulated by the C . elegans transmembrane protein FLD-1 and its human homologs TLCD1 / 2. Elife. 2018;1–25.

48. Jacobsen KT, Adlerz L, Multhaup G, Iverfeldt K. Insulin-like Growth Factor-1 (IGF-1) -induced Processing of Amyloid- Precursor Protein (APP) and APP-like Protein 2 Is Mediated by Different Metalloproteinases. J Biol Chem. 2010;285(14):10223–31.

49. He T, Katusic ZS. Expression and Processing of Amyloid Precursor Protein in Vascular. PHYSIOLOGY. 2017;32(94):20–32.

50. Miao L, Yin RX, Pan SL, Yang S, Yang DZ, Lin WX. Weighted Gene Co-Expression Network Analysis Identifies Specific Modules and Hub Genes Related to Hyperlipidemia. Cell Physiol Biochem. 2018;48(3):1151–63.

51. Printen JA, Brady MJ, Saltiel AR. PTG , a Protein Phosphatase 1 – Binding Protein with a Role in Glycogen Metabolism. 1997;275(5305):1475–9.

52. Hiruma T, Togayachi A, Okamura K, Sato T, Kikuchi N, Kwon Y-D, et al. A Novel Human E 1 , 3- N - Acetylgalactosaminyltransferase Which Synthesizes a Unique Carbohydrate Structure , GalNAc E 1-3GlcNAc *. J Biol Chem. 2004;279(14):14087–95.

53. Lee PL, Kohler JJ, Pfeffer SR. Association of β -1 , 3- N -acetylglucosaminyltransferase 1 and β -1 , 4-

galactosyltransferase 1 , trans- Golgi enzymes involved in coupled poly- N -acetyllactosamine synthesis. 2009;19(6):655–64.

54. DAVID Bioinformatics Resources 6.8 Laboratory of Human Retrovirology and Immunoinformatics (LHRI).

55. Pérez VI, Lew CM, Cortez LA, Webb CR, Rodriguez M, Liu Y, et al. Thioredoxin 2 haploinsufficiency in mice results in impaired mitochondrial function and increased oxidative stress. Free Radic Biol Med. 2008;44(5):882–92.

56. Liu D, Liu Y, Xia Z, Dong H, Yi Z. Reactive oxygen species modulator 1 regulates oxidative stress and induces renal and pulmonary fibrosis in a unilateral ureteral obstruction rat model and in HK-2 cells. Mol Med Rep. 2017;16(4):4855–62.

57. Lee SH, Park MJ, Choi SI, Lee EJ, Lee SY, In KH. Reactive oxygen species modulator 1 (Romo1) as a novel diagnostic marker for lung cancer-related malignant effusion. Med (United States). 2017;96(4):1–5.

58. Hou R, Sibinga NES. Atrophin proteins interact with the Fat1 cadherin and regulate migration and orientation in vascular smooth muscle cells. J Biol Chem. 2009;284(11):6955–65.

59. Rojanathammanee L, Rakoczy S, Brown-Borg HM. Growth hormone alters the glutathione S-transferase and mitochondrial thioredoxin systems in long-living ames dwarf mice. Journals Gerontol - Ser A Biol Sci Med Sci. 2014;69(10):1199–211.

60. Grabež V, Kathri M, Phung V, Moe KM, Slinde E, Skaugen M, et al. Protein expression and oxygen consumption rate of early postmortem mitochondria relate to meat tenderness. J Anim Sci. 2015;93(4):1967–79.

61. Pader I, Sengupta R, Cebula M, Xu J, Lundberg JO, Holmgren A, et al. Thioredoxin-related protein of 14 kDa is an efficient L-cystine reductase and S-denitrosylase. Proc Natl Acad Sci [Internet]. 2014;111(19):6964–9.

62. Hussain T, Al-Attas OS, Al-Daghri NM, Mohammed AA, De Rosas E, Ibrahim S, et al. Induction of CYP1A1, CYP1A2, CYP1B1, increased oxidative stress and inflammation in the lung and liver tissues of rats exposed to incense smoke. Mol Cell Biochem. 2014;391(1–2):127–36.

63. Bruder-Nascimento T, Chinnasamy P, Riascos-Bernal DF, Cau SB, Callera GE, Touyz RM, et al. Angiotensin II induces Fat1 expression/activation and vascular smooth muscle cell migration via Nox1-dependent reactive oxygen species generation. J Mol Cell Cardiol. 2014;66:18–26.

64. Schulman S, Bendapudi P, Sharda A, Chen V, Bellido-Martin L, Jasuja R, et al. Extracellular Thiol Isomerases and Their Role in Thrombus Formation. Antioxid Redox Signal [Internet]. 2016;24(1):1–15.

65. Boison D. Adenosine Kinase: Exploitation for Therapeutic Gain. Pharmacol Rev [Internet]. 2013;65(3):906–43.

66. Anasa VV, Ravanan P, Talwar P. Multifaceted roles of ASB proteins and its pathological significance. Front Biol (Beijing). 2018;13(5):376–88.

67. D'Ippolito AM, McDowell IC, Barrera A, Hong LK, Leichter SM, Bartelt LC, et al. Pre-established Chromatin Interactions Mediate the Genomic Response to Glucocorticoids. Cell Syst [Internet]. 2018;7(2):146–160.e7.

68. Di Taranto MD, Morgante A, Bracale UM, D'Armiento FP, Porcellini M, Bracale G, et al. Altered expression of inflammation-related genes in human carotid atherosclerotic plaques. Atherosclerosis [Internet]. 2012;220(1):93–101.

69. Bouças AP, Oliveira F dos S de, Canani LH, Crispim D. The role of interferon induced with helicase C domain 1 (IFIH1) in the development of type 1 diabetes mellitus. Arq Bras Endocrinol Metabol [Internet]. 2013;57(9):667–76.

70. Viemann D, Goebeler M, Schmid S, Nordhues U, Klimmek K, Sorg C, et al. TNF induces distinct gene expression programs in microvascular and macrovascular human endothelial cells. J Leukoc Biol [Internet]. 2006;80(1):174–85.

71. Tiret L, Godefroy T, Lubos E, Nicaud V, Tregouet DA, Barbaux S, et al. Genetic analysis of the interleukin-18 system highlights the role of the interleukin-18 gene in cardiovascular disease. Circulation. 2005;112(5):643–50.

72. Mendiratta SK, Martin WD, Hong S, Boesteanu A, Joyce S, Van Kaer L. CD1d1 mutant mice are deficient in natural T cells that promptly produce IL-4. Immunity. 1997;6(4):469–77.

73. Neerincx A, Rodriguez GM, Steimle V, Kufer TA. NLRC5 Controls Basal MHC Class I Gene Expression in an MHC Enhanceosome-Dependent Manner. J Immunol [Internet]. 2012;188(10):4940–50.

74. Fotouhi-Ardakani N, El Kebir D, Pierre-Charles N, Wang L, Ahern SP, Filep JG, et al. Role for myeloid nuclear differentiation antigen in the regulation of neutrophil apoptosis during sepsis. Am J Respir Crit Care Med. 2010;182(3):341–50.

75. Lin HY, Yin Y, Zhang JX, Xuan H, Zheng Y, Zhan SS, et al. Identification of direct forkhead box O1 targets involved in palmitate-induced apoptosis in clonal insulin-secreting cells using chromatin immunoprecipitation coupled to DNA selection and ligation. Diabetologia. 2012;55(10):2703–12.

76. Fischer H, Scherz J, Szabo S, Mildner M, Benarafa C, Torriglia A, et al. Dnase 2 is the main DNA-degrading enzyme of the stratum corneum. PLoS One. 2011;6(3).

77. Scott DD, Trahan C, Zindy PJ, Aguilar LC, Delubac MY, Van Nostrand EL, et al. Nol12 is a multifunctional RNA binding protein at the nexus of RNA and DNA metabolism. Nucleic Acids Res. 2017;45(21):12509–28.

78. Cannavo A, Liccardo D, Komici K, Corbi G, de Lucia C, Femminella GD, et al. Sphingosine kinases and sphingosine 1-phosphate receptors: Signaling and actions in the cardiovascular system. Front Pharmacol. 2017;8(AUG):1–12.

79. Roy S, Kuruc M. Methods to Monitor the Functional Subproteomes of SERPIN Protease Inhibitors. Methods Mol Biol. 2018;1871:41–54.

80. Ito K, Takahashi A, Morita M, Suzuki T, Yamamoto T. The role of the CNOT1 subunit of the CCR4-NOT complex in mRNA deadenylation and cell viability. Protein Cell. 2011;2(9):755–63.

81. Drosatos K. Fatty old hearts: Role of cardiac lipotoxicity in age-related cardiomyopathy. Pathobiol Aging Age-related Dis. 2016;6.

82. Kohroki J, Nishiyama T, Nakamura T, Masuho Y. ASB proteins interact with Cullin5 and Rbx2 to form E3 ubiquitin ligase complexes. FEBS Lett. 2005;579(30):6796–802.

83. Zhang A, He X, Zhang L, Yang L, Woodman P, Li W. Biogenesis of lysosome-related organelles complex-1 subunit 1 (BLOS1) interacts with sorting nexin 2 and the endosomal sorting complex required for transport-I (ESCRT-I) component TSG101 to mediate the sorting of epidermal growth factor receptor into endo. J Biol Chem. 2014;289(42):29180–94.

84. Moretti F, Bergman P, Dodgson S, Marcellin D, Claerr I, Goodwin JM, et al. TMEM41B is a novel regulator of autophagy and lipid mobilization. EMBO Rep [Internet]. 2018;e45889.

85. Yen WL, Shintani T, Nair U, Cao Y, Richardson BC, Li Z, et al. The conserved oligomeric Golgi complex is involved in double-membrane vesicle formation during autophagy. J Cell Biol. 2010;188(1):101–14.

86. Kitamura H, Matsumori H, Kalendova A, Hozak P, Ilya G, Nakao M, et al. The actin family protein ARP6 contributes to the structure and the function of the nucleolus. Biochem Biophys Res Commun. 2015;464(2):1–15.

87. Yu L, Wang Y, Huang S, Wang J, Deng Z, Zhang Q, et al. Structural insights into a novel histone demethylase PHF8. Cell Res. 2010;20(2):166–73.

88. Jilani A, Ramotar D, Slack C, Ong C, Yang XM, Scherer SW, et al. Molecular cloning of the human gene, PNKP, encoding a polynucleotide kinase 3'-phosphatase and evidence for its role in repair of DNA strand breaks caused by oxidative damage. J Biol Chem. 1999;274(34):24176–86.

89. Vannier JB, Sarek G, Boulton SJ. RTEL1: Functions of a disease-associated helicase. Trends Cell Biol [Internet]. 2014;24(7):416–25.

90. Poli J, Gasser SM, Papamichos-Chronakis M. The INO80 remodeller in transcription, replication and repair. Philos Trans R Soc B Biol Sci. 2017;372(1731).

91. Huang P, Cai Y, Zhao B, Cui L. Roles of NUCKS1 in Diseases: Susceptibility, Potential Biomarker, and Regulatory

Mechanisms. Biomed Res Int. 2018;2018.

92. Krem MM, Luo P, Ing BI, Horwitz MS. The kelch protein KLHDC8B guards against mitotic errors, centrosomal amplification, and chromosomal instability. J Biol Chem. 2012;287(46):39083–93.
